# New Functionalized Phenoxazines and Phenothiazines

**DOI:** 10.1021/acsomega.3c06461

**Published:** 2023-11-09

**Authors:** M. John Plater, William T. A. Harrison

**Affiliations:** Department of Chemistry, University of Aberdeen, Meston Walk, Aberdeen AB24 3UE, United Kingdom of Great Britain and Northern Ireland

## Abstract

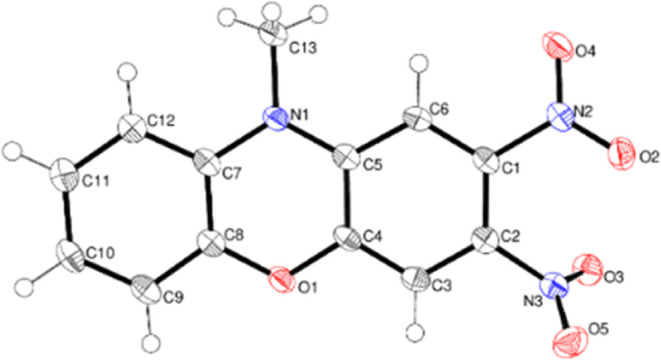

The reaction of either 2-aminophenol or 2-(*N*-methylamino)phenol with 1,2-difluoro-4,5-dinitrobenzene and sodium carbonate in EtOH gives 2,3-dinitrophenoxazines. One nitro group, conjugated to the aryl ether, was displaced from 2,3-dinitro-10-methylphenoxazine with different nucleophiles: BuNH_2_, KOEt, and KOH. The reaction of 2-aminothiophenol with 1,2-difluoro-4,5-dinitrobenzene under the same conditions gives 2,3-dinitrophenothiazine. This reacted with BuNH_2_ forming 2-butylamino-3-nitrophenothiazine. The dihedral angles of the different compounds are compared.

## Introduction

**Figure 1:**
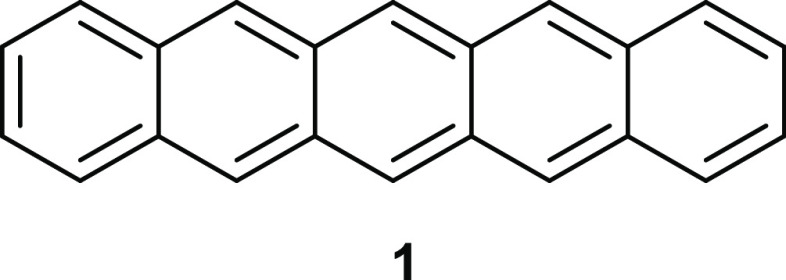
Pentacene.

**Figure 2:**
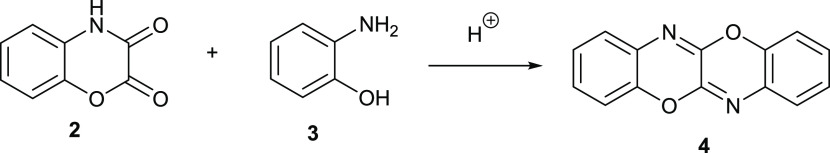
Heterocyclic derivative of tetracene.

**Figure 3:**
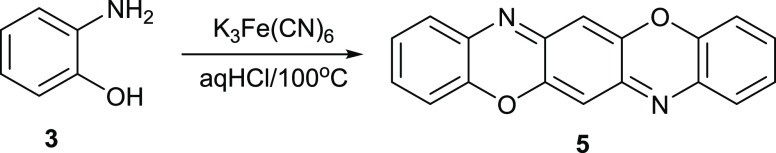
Heterocyclic derivative of pentacene for potential material applications.^28^

**Figure 4:**
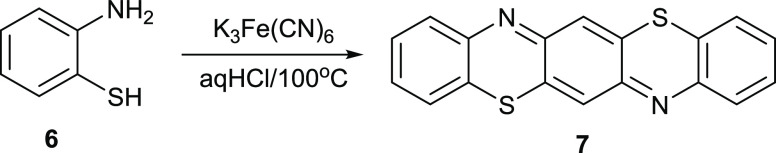
Heterocyclic derivative of pentacene for potential material applications.^28^

Pentacene **1** has been investigated in organic electronics and device physics^1,2^ with fundamental studies on electronic^3−7^ and optical^8−11^ properties (Figure 1). However, it suffers from long-term stability because of its photo-oxidation and low thermal stability.^12^ To overcome these drawbacks and to provide alternative molecules with improved film stability, morphology, and processing properties, heteroatom-containing derivatives of pentacene, broadly known as N-heteroacenes^13−15^ and NOS-heteroacenes,^16−18^ have been investigated. There is much early work on the heterocyclic analogues of pentacene and related compounds (Figures 2–4).^19−25^

Figure 2 shows a one-step heterocyclic synthesis of an analogue of tetracene using compound **2** and 2-aminophenol **3**.^26^

Figure 3 shows a one-pot oxidation of 2-aminophenol **3** with K_3_Fe(CN)_6_ in dilute HCl under reflux^27,28^ to give a heterocyclic analogue of pentacene **5**.^27,28^ The clean product crystals were filtered off from the aqueous system. This method and others give a product in which the polar amino and hydroxyl groups are condensed into the ring system or the middle part is capped on both ends. Further methods for making compound **5** involve the FeCl_3_ oxidation of phenoxazine followed by reaction with 2-aminophenol **3**,^29^ 2-aminophenol **3** with hydroxybenzoquinone,^30^ benzoquinone,^31^ 2,5-dibromobenzoquinone,^32^ and dichloronaphthoquinone.^33^

A similar method for making compound **5** has been reported for making compound **7** and its derivatives for organic electroluminescent materials and optoelectronic devices (Figure 4).^28^ Materials in this field are often exploited as organic transistors^34−38^ and light-emitting diodes.^39,40^

## Results and Discussion

**Figure 5:**
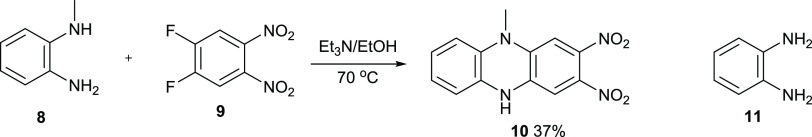
4,5-Difluoro-1,2-dinitrobenzene **9** for phenazine heterocyclic synthesis.

Previously, we reported the synthesis of the poorly stable phenazine derivative **10** using 4,5-difluoro-1,2-dinitrobenzene **9**.^41,42^ This reaction failed with *ortho*-phenylenediamine **11** but worked with *N*-methyl-*o*-phenylenediamine **8** because the alkylated amine is more nucleophilic, which gets the reaction started followed by a cyclization (Figure 5).

**Figure 6:**
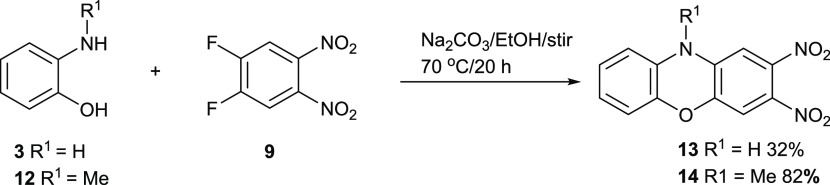
Synthesis of dinitrophenoxazines **13** and **14**.

This article reports the first synthesis of phenoxazine and phenothiazine derivatives using 4,5-difluoro-1,2-dinitrobenzene **9**. Poorly soluble heterocyclic acenes are difficult to make and characterize, so the compounds prepared in this paper are smaller and easier to work with. Figure 6 shows a reaction of 2-hydroxyaniline **3** and 2-hydroxy-*N*-methylaniline **12** with building block **9** in hot ethanol with Na_2_CO_3_ as a base. Phenoxazine **13** was formed in a lower yield of 32%, and methylated phenoxazine **14** was formed in a higher yield of 82%. The difference in yield suggests that the mechanistic pathway for these two reactions may be different. This is explained below.

The use of Na_2_CO_3_ as a stirred suspension of base is similar to the Williamson ether synthesis using K_2_CO_3_ stirred in acetone to alkylate phenols.^43^ For compound **3**, the phenol as phenoxide probably displaces fluoride first, followed by a fast intramolecular cyclization of the primary amine. For compound **12**, the more nucleophilic *N*-methylamine probably displaces fluoride first, followed by a fast intramolecular cyclization of the phenol as phenoxide.

**Figure 7:**
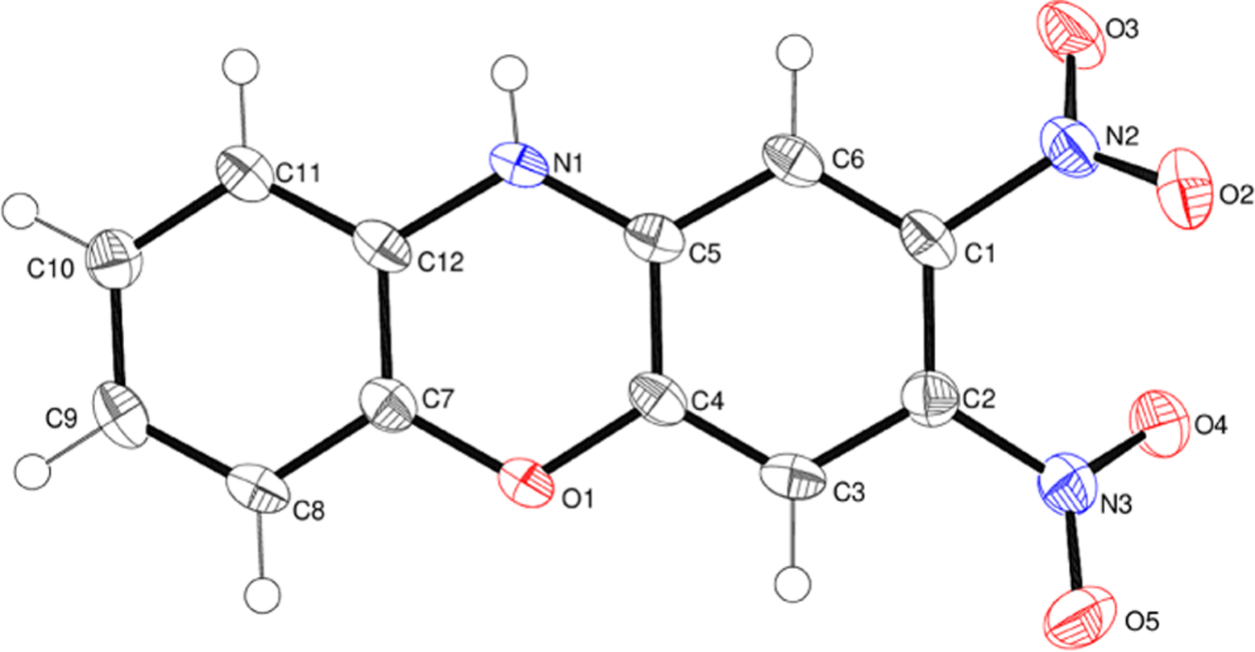
Molecular structure of compound **13** showing 50% displacement ellipsoids.

Compound **13** crystallizes in the orthorhombic space group *P*2_1_2_1_2_1_ with one molecule in the asymmetric unit (Figure 7). The fused-ring system is slightly puckered, and the dihedral angle between the C1–C6 and C7–C12 rings is 3.027 (14)°. Both the N2 and N3 nitro groups are significantly twisted away from the C1–C6 ring plane [dihedral angles = 43.64 (17)° and 31.67 (17)°, respectively], presumably to minimize steric repulsion between them [the dihedral angle between the nitro groups is 50.6 (5)°]. The C1–C6 and C3–C4 bond lengths (mean = 1.370 Å) are shorter than C5–C6 and C2–C3 (mean = 1.394 Å), possibly indicating some conjugation of the N1 lone pair electrons with the ring, and C2–N3 [1.450 (5) Å] is shorter than C1–N2 [1.469 (5) Å] for the same reason, although the effect is quite small. In the extended structure of **13**, weak bifurcated N–H···(O,O) hydrogen bonds to the O atoms in the N2 nitro group link the molecules into [010] chains.

**Figure 8:**
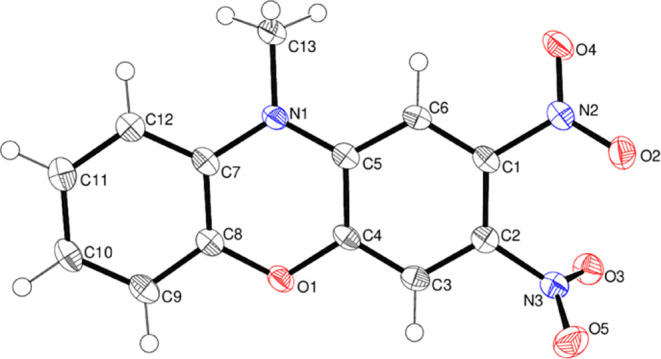
Molecular structure of compound **14** showing 50% displacement ellipsoids.

The molecular structure of compound **14**, which also crystallizes in the space group *P*2_1_2_1_2_1_ with one molecule in the asymmetric unit, is shown in Figure 8. Its fused-ring system shows significantly more puckering than that in **13**, with a dihedral angle of 10.461 (16)° between the C1 and C7–C12 rings. The central phenoxazine ring is well described as a shallow boat, with N1 and O1 displaced from the mean plane of C4/C5/C7/C8 (r.m.s. deviation = 0.002 Å) by −0.161 (2) and −0.147 (2) Å, respectively. The N2 nitro group is rotated from its attached ring by 44.08 (9)°, and the N3 nitro group is even more twisted [dihedral angle = 76.64 (15)°]: the dihedral angle between the nitro groups is 46.05 (15)°. In the extended structure of **14**, some weak C–H···O hydrogen bonds may help to consolidate the packing.

**Figure 9:**
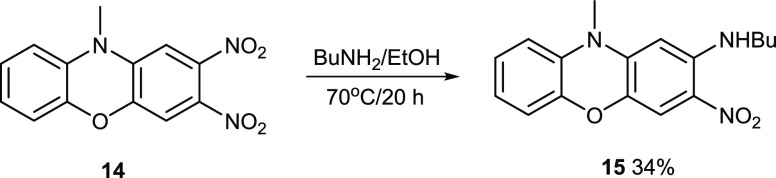
Synthesis of compound **15**.

Two *ortho* nitro groups activate each other to nucleophilic displacement.^44^ So far, in this synthesis, the fluoride groups are displaced first before the nitro groups. However, Figure 9 shows the selective displacement of just one of the *ortho* nitro groups with butylamine. The 2-nitro group is the most activated, and it is *para* to the aryl ether. The 3-nitro group is less easily displaced because it is conjugated to the electron-rich nitrogen lone pair.

**Figure 10:**
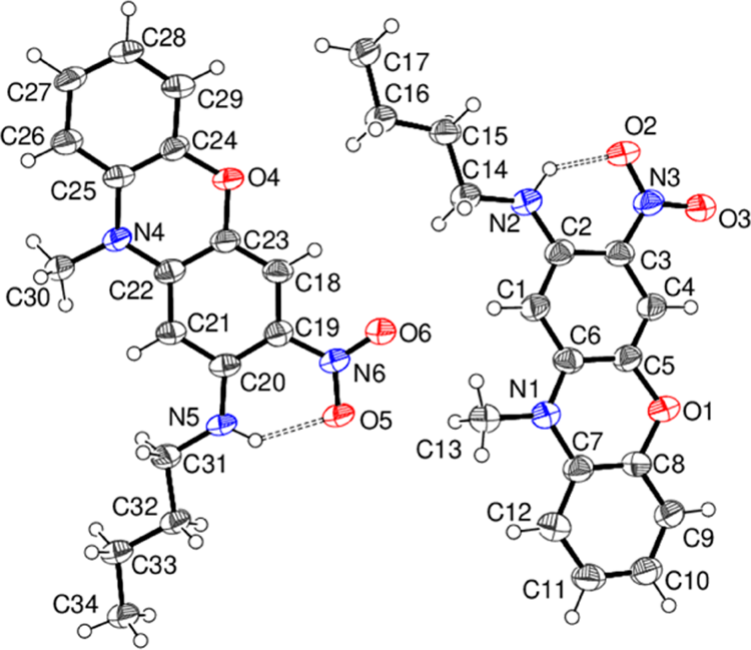
Molecular structure of compound **15** showing 50% displacement ellipsoids. The N–H···O hydrogen bonds are represented by double-dashed lines.

The crystal structure of compound **15**, which was established from synchrotron data (Figure 10), shows the presence of two molecules (A containing C1 and B containing C18) in the asymmetric unit in the triclinic space group *P*1̅. The molecules have broadly similar conformations although the fused-ring system in A [dihedral angle between the outer rings = 10.08 (14)°] is rather more puckered than in B [4.93 (15)°]. The nitro group is close to the plane of its attached ring in both molecules [dihedral angles = 3.2 (2) and 6.9 (2)° for molecules A and B, respectively] and the *n*-butyl chain adopts an extended conformation in both A and B. In both molecules, an intramolecular N–H···O hydrogen bond closes an *S*(6) ring with H···O = 1.82 (4) Å and N–H···O = 142 (4)° for molecule A with equivalent data of 1.97 (4) Å and 139 (4)°, respectively, for molecule B. Some weak intermolecular C–H···O hydrogen bonds may help to consolidate the extended structure of **15**.

**Figure 11:**
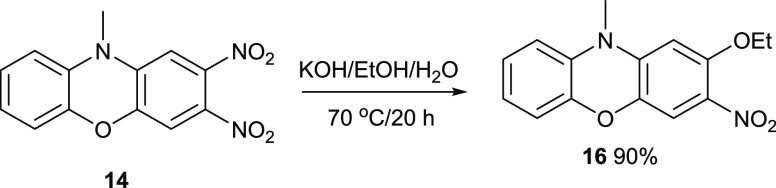
Synthesis of compound **16**.

Not surprisingly, other nucleophiles displaced the 2-nitro group like butylamine did. Treatment of *N*-methyldinitrophenoxazine **14** with KOH/EtOH/H_2_O gave a high yield of compound **16** (Figure 11). TLC analysis showed no displacement by hydroxide to have occurred. This result was unexpected, as water is more acidic than EtOH and was expected to displace the fluoride atom. One explanation could be deactivation of the two nitro groups by the electron-rich benzene ring, so that an equilibrium amount of ethoxide is required for the reaction.

**Figure 12:**
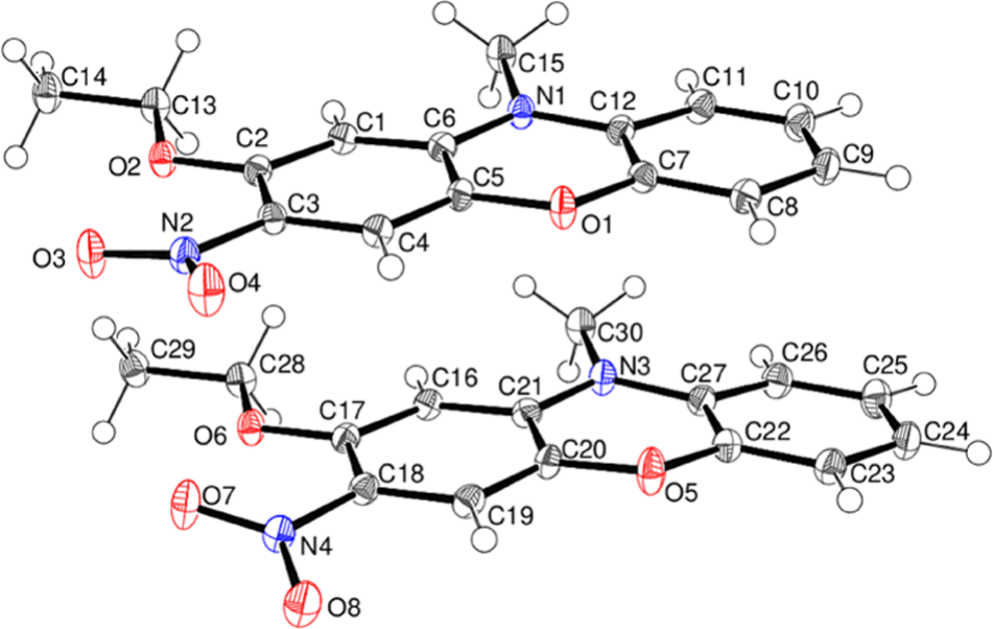
Molecular structure of compound **16** showing 50% displacement ellipsoids.

Compound **16** crystallizes with two molecules (A containing C1 and B containing C16) in the asymmetric unit (Figure 12) in the monoclinic space group *P*2_1_/*n*. Their conformations are similar to a dihedral angle of 2.74 (2)° between the C1–C6 and C7–C12 rings in molecule A and an equivalent angle of 6.372 (14)° for the C16–C21 and C22–C27 rings in molecule B. The plane of the nitro group lies close to its attached ring in both molecules [dihedral angles = 6.98 (5) and 17.08 (5)° for molecules A and B, respectively], and the C2–O2–C13-C14 and C17–O6–C28–C29 torsion angles are −173.24 (8) and −174.01 (8)°, respectively. In the extended structure of compound **16**, some weak C–H···O hydrogen bonds link the molecules.

**Figure 13:**
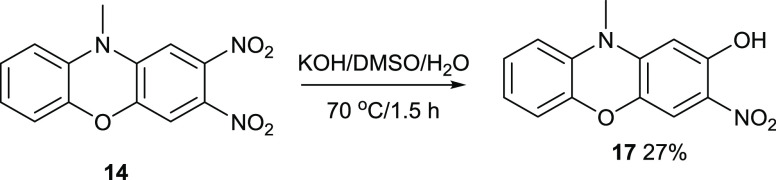
Synthesis of compound **17**.

Since in mixtures of KOH/EtOH/H_2_O, KOEt acted as a nucleophile toward compound **14**, KOH/DMSO/H_2_O was used to favor KOH as a nucleophile (Figure 13). DMSO has a much higher p*K*_a_ than EtOH, so KOH is more likely to be the soluble base. This proved to be the case, but the yield of compound **17** is much lower.

**Figure 14:**
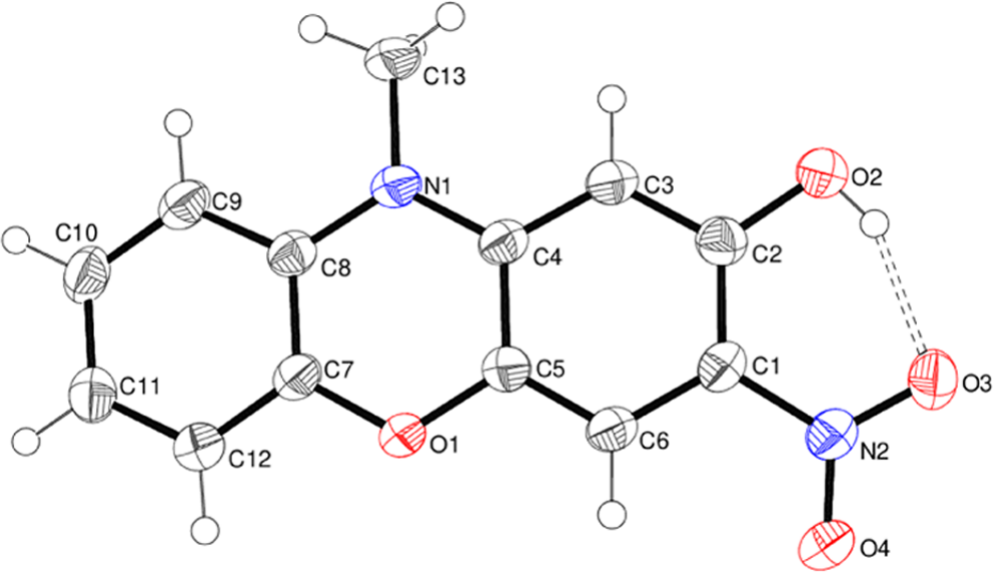
Molecular structure of compound **17** showing 50% displacement ellipsoids. The O–H···O hydrogen bond is indicated by a double-dashed line.

Compound **17** crystallizes with one molecule in the asymmetric unit in the triclinic space group *P*1̅ (Figure 14). The dihedral angle between the outer rings is 4.23 (9)°, and the heterocyclic ring is a very shallow boat, with N1 and O1 displaced by 0.054 (4) and 0.070 (3) Å, respectively, from the four carbon atoms (rms deviation = 0.014 Å). The N2 nitro group is rotated by 6.7 (3)° from its attached ring, and an intramolecular O–H···O hydrogen bond closes an *S*(6) ring with H···O = 1.84 (3) Å and O–H···O = 154 (3)°. Some weak C–H···O hydrogen bonds occur in the extended structure.

**Figure 15:**
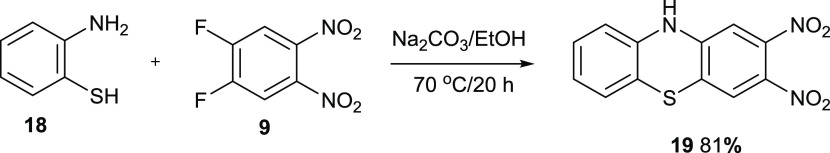
Synthesis of compound **19**.

Thiols and thiolate anions are good nucleophiles, so we expected 2-aminothiophenol **18** to react efficiently with compound **9** provided the fluorine atoms displace first (Figure 15). The thiophenazine heterocycle **19** forms in an 81% yield. Presumably, the thiol or thiolate anion displaces fluorine first followed by an intramolecular cyclization of the primary amine.

**Figure 16:**
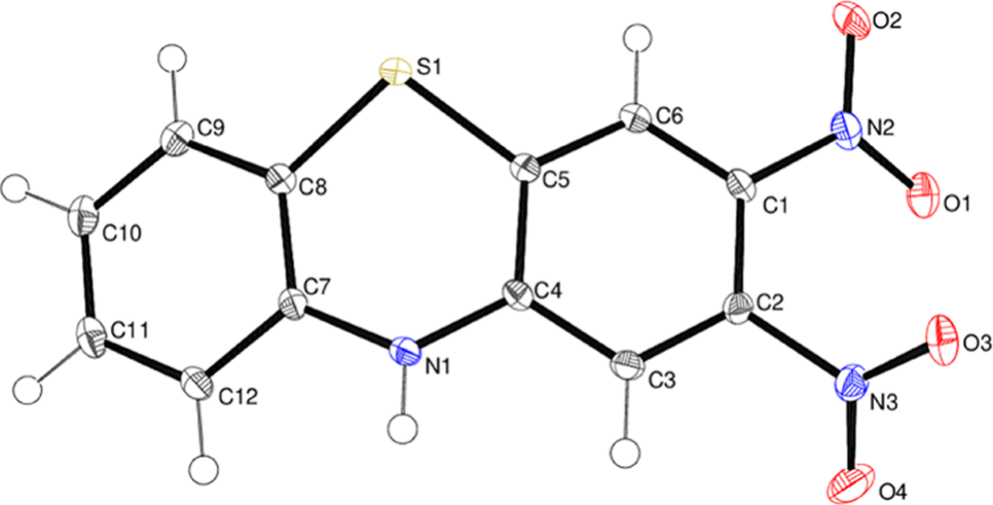
Molecular structure of compound **19** showing 50% displacement ellipsoids.

Compound **19** (Figure 16) crystallizes with one molecule in the asymmetric unit in the space group *P*2_1_2_1_2_1_. The phenothiazine fused-ring system in compound **19** is significantly puckered compared to those of the phenoxazines described above, with a dihedral angle of 23.13 (3)° between the C1–C6 and C7–C12 rings. This compares with a dihedral angle of 21.5° between the outer rings in phenothiazine, C_12_H_8_NS.^45,46^ The central heterocycle in compound **19** is a distorted boat with atoms N1 and S1 displaced by −0.245 (2) and −0.503 (2) Å, respectively, from C4/C5/C7/C8 (rms deviation = 0.007 Å). The N2 and N3 nitro groups are rotated by 21.02 (11) and 51.63 (6)°, respectively, with respect to their attached ring. In the crystal, a N–H···O hydrogen bond with H···O = 2.26 (2) Å and N–H···O = 166 (2)° generates [001] chains.

**Figure 17:**
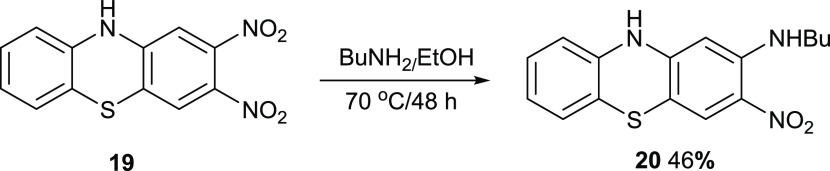
Synthesis of compound **20**.

The 2-nitro group can be displaced with butylamine as for compound **14**, but the heterocyclic ring system is more robust, so the reaction required 48 h (Figure 17).

**Figure 18:**
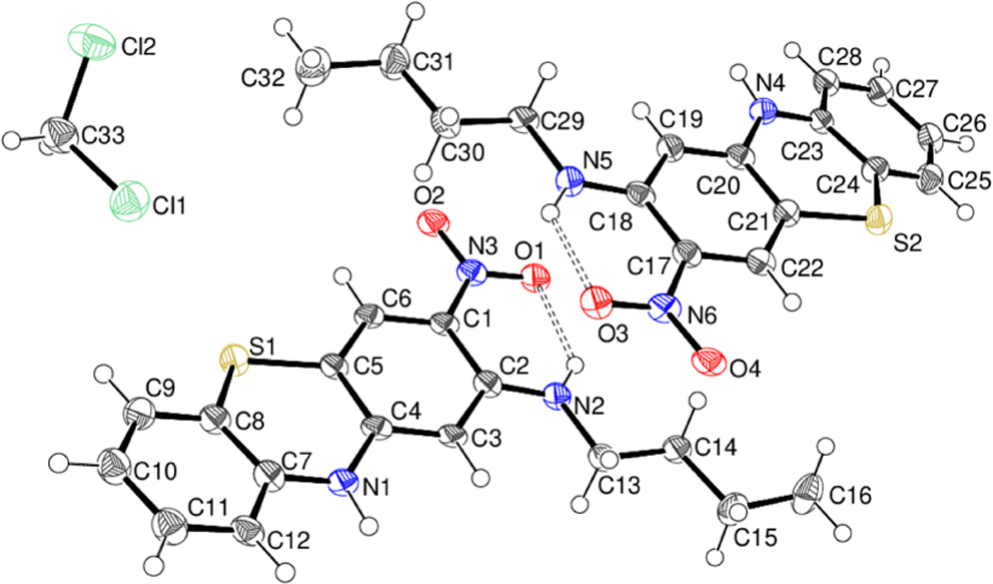
Molecular structure of compound **20** showing 50% displacement ellipsoids. The N–H···O hydrogen bonds are indicated by double-dashed lines.

Compound **20** crystallizes with two phenothiazine molecules (A containing C1 and B containing C17) in the asymmetric unit accompanied by a dichloromethane solvent molecule (Figure 18) in the monoclinic space group *P*2_1_/*c*. The molecules have similar conformations, with a dihedral angle between the outer rings of 14.2 (3)° for A and 13.6 (3)° for B. The near-coplanarity of the nitro group with its attached ring [dihedral angles = 1.9 (6) and 1.7 (6)° for molecules A and B, respectively] is reinforced by the formation of an intramolecular N–H···O hydrogen bond with H···O = 1.97 Å for A and 1.95 Å for B and N–H···O = 131 and 130°, respectively. In the extended structure of compound **20**, N–H···O hydrogen bonds link the molecules into [010] chains, which are reinforced by weak C–H···O links. The solvent molecules occupy small [100] channels.

## Conclusions

The synthetic use of 1,2-difluoro-4,5-dinitrobenzene **9** for making heterocycles has been extended. 2,3-Dinitrophenoxazine **13** and 2,3-dinitrophenothiazine **19** are easily formed by reacting 2-aminophenol **3** or 2-aminothiophenol **18**, respectively, with 1,2-difluoro-4,5-dinitrobenzene **9** using Na_2_CO_3_ as a base in hot EtOH. The use of 2-hydroxy-*N*-methylaniline **12** gave phenoxazine **14** in a high yield of 80%, presumably because the *N*-alkyl amino group is more nucleophilic, compared to aniline, and displaces fluorine rapidly. The two nitro groups of phenazine **14** or phenothiazine **19** activate each other, and one nitro group can be displaced with butylamine to give phenazine **15** and phenothiazine **20**, respectively. The nitro group *para* to the aryl ether is displaced in preference to the one *para* to the more electron-rich amino group. Phenoxazine **14** gives an unusual reaction with KOH/EtOH/H_2_O, forming 2-ethoxy-3-nitro-10-methylphenoxazine **16**. An equilibrium concentration of ethoxide must displace the 2-nitro group rather than hydroxide. With KOH/DMSO/H_2_O, hydroxide displaces the 2-nitro group from phenoxazine **14** as expected to give 2-hydroxy-3-nitro-10-methylphenoxazine **17**. The dihedral angles for each compound are compared. For 2,3-dinitrophenothiazine, it is 23°, which is larger than that for the phenoxazines studied. These fall in the range of 3–10°.

## Experimental Section

IR spectra were recorded on a thermoscientific Nicolet Summit diamond-attenuated total reflection (ATR) Fourier transform infrared (FTIR) spectrometer. Ultraviolet (UV) spectra were recorded by using a PerkinElmer Lambda 25 UV–vis spectrometer with EtOH as the solvent. The term “sh” means shoulder. ^1^H and ^13^C nuclear magnetic resonance (NMR) spectra were recorded at 400 and 100.5 MHz, respectively, by using a Bruker 400 spectrometer. Chemical shifts, δ, are given in ppm and measured by comparison with the residual solvent. Coupling constants, *J*, are given in Hz. A broad signal is abbreviated as br. High-resolution mass spectra were obtained at the University of Wales, Swansea, using an Atmospheric Solids Analysis Probe (ASAP) (positive mode) instrument: Xevo G2-S ASAP. Melting points were determined on a U.K. Cole-Palmer Stuart microscope.
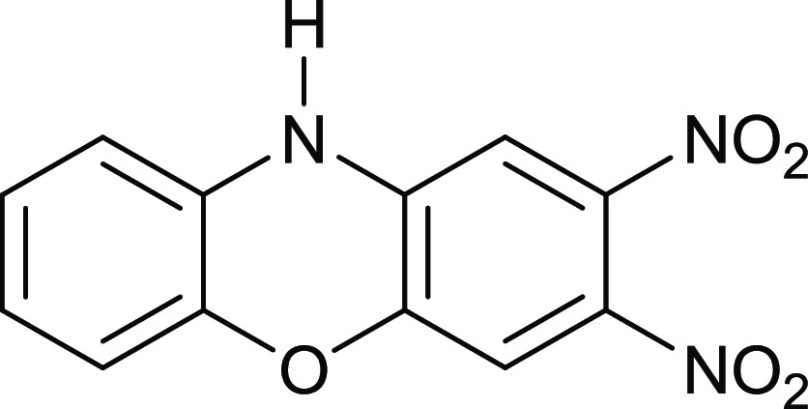


### 2,3-Dinitro-10H-phenoxazine **13**

4,5-Difluoro-1,2-dinitrobenzene (100 mg, 0.49 mmol) in EtOH (30 mL) was mixed with 2-aminophenol (54 mg, 0.49 mmol) and Na_2_CO_3_ (500 mg) and then stirred at 70 °C for 6 h. After cooling, the mixture was added to water (200 mL), left standing for 1 h, filtered through a sinter grade No. 4, and air-dried to give the *title compound* (43 mg, 32%) as pure red crystals; mp >245 °C (from dichloromethane:light petroleum ether). λ_max_ (EtOH)/nm 283 (log ε 4.3), 378 (3.8) and 468 (3.9); ν_max_ (diamond)(cm^–1^) 3362w, 1633w, 1575s, 1514s, 1494s, 1450s, 1396s, 1314s, 1208s, 869s, 826s, 746s and 491s; δ_H_ (400 MHz; D_7_DMF) 6.80 (1H, d, *J* = 8.0), 6.89 (1H, d, *J* = 8.0), 6.97 (1H, t, *J* = 8.0 and 8.0), 7.04 (1H, t, *J* = 8.0 and 8.0), 7.12 (1H, s) and 7.51 (1H, s); δ_C_ (100.1 MHz; D_7_DMF) 108.0, 112.1, 115.2, 116.0, 123.8, 125.5, 129.3, 132.9, 140.0, 142.2, 142.7 and 144.9; *m*/*z* (Orbitrap ASAP) 274.0459 (M^+^ + H) C_12_H_7_N_3_O_5_H requires 274.0464.
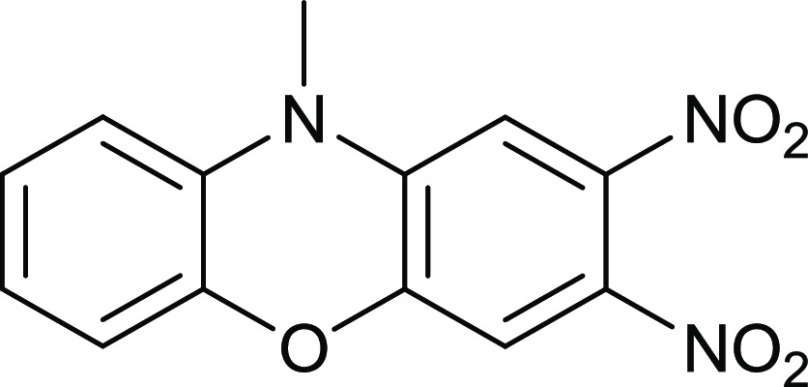


### 2,3-Dinitro-*N*-methylphenoxazine **14**

4,5-Difluoro-1,2-dinitrobenzene (332 mg, 1.6 mmol) in EtOH (30 mL) was mixed with 2-(methylamino)phenol (200 mg, 1.6 mmol) and Na_2_CO_3_ (2.0 g) and then stirred at 70 °C for 20 h. After cooling, the mixture was added to water (200 mL), left standing for 1 h, filtered through a sinter grade No. 4, and air-dried to give the *title compound* (383 mg, 82%) as pure red crystals, mp 164–165 °C (from dichloromethane:light petroleum ether). λ_max_ (EtOH)/nm 283 (log ε 3.9), 373 (3.4) and 463 (3.5); ν_max_ (diamond)(cm^–1^) 3091w, 1525s, 1491s, 1370s, 1329s, 1293s, 1220, 1202, 1064s, 870s, 824s and 741s; δ_H_ (400 MHz; CDCl_3_) 3.14 (3H, s), 6.67 (3H, d, *J* = 8.0), 6.91 (2H, *J* = 8.0) and 7.24 (1H, d, *J* = 8.0); δ_C_ (100.1 MHz; CDCl_3_) 31.8, 106.2, 111.6, 112.9, 116.1, 124.0, 125.4, 131.1, 134.6, 139.9, 141.2, 143.9 and 146.9; *m*/*z* (Orbitrap ASAP) 288.0612 (M^+^ + H, 100%) C_13_H_9_N_3_O_5_H requires 288.0620.
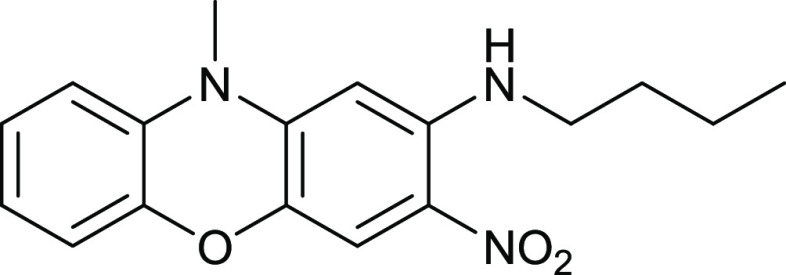


### 2-Butylamino-3-nitro-*N*-methylphenoxazine **15**

*N*-Methyl-3,4-dinitrophenoxazine (50 mg, 0.17 mmol) in EtOH (30 mL) was mixed with butylamine (61 mg, 0.69 mmol) and heated at 70 °C for 20 h. After cooling, the mixture was mixed with water (170 mL) and filtered, and the precipitate was purified by chromatography. Elution with DCM gave the *title compound* (18 mg, 34%) as red crystals, mp 147–148 °C (from dichloromethane). λ_max_ (EtOH)/nm 244 (log ε 3.2), 284 (2.6), 394 (2.5) and 484 (2.8); ν_max_ (diamond)(cm^–1^) 2956w, 2931w, 2865w, 1939s, 1608s, 1575s, 1484s, 1470s, 1400s, 1335s, 1248s, 1225s, 1195s, 1169s, 1041s and 733s; δ_H_ (400 MHz; D_7_DMF) 0.97 (3H, t, *J* = 7.0), 1.46 (2H, s, *J* = 7.0), 1.72 (2H, q, *J* = 7.0), 3.35 (3H, s), 3.46 (2H, q, *J* = 7.0), 6.12 (1H, s), 6.86 (1H, d, *J* = 8.0), 6.92 (1H, t, *J* = 8.0 and 8.0), 6.98–7.07 (2H, m), 7.33 (1H, s) and 8.69 (1H, s, br, NH); δ_C_ (100.1 MHz; D_7_DMF) 13.3, 20.1, 30.9, 31.5, 42.4, 93.6, 109.1, 113.9, 115.6, 122.4, 123.2, 124.1, 130.8, 135.6, 142.9, 144.1, and 146.4; *m*/*z* (Orbitrap ASAP) 314.1510 (M^+^ + H, 100%) C_17_H_19_N_3_O_3_H requires 314.1505.
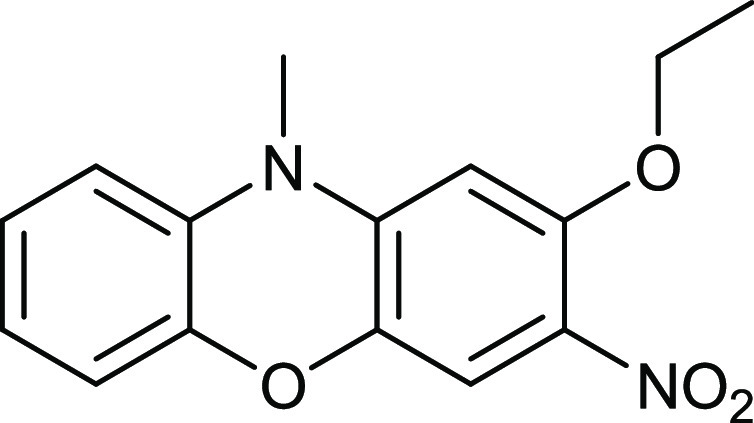


### 2-Ethoxy-3-nitro-*N*-methylphenoxazine **16**

(20 mg, 0.0697 mmol) was mixed with EtOH (18 mL) and an aqueous base (90 mg of KOH in 1 mL of water). The mixture was heated at 70 °C for 20 h with stirring. After cooling, the mixture was neutralized with dilute hydrochloric acid (5 mL of cHCl in 100 mL water) and then filtered to give the *title compound* (18 mg, 90%) as orange crystals; mp 175–176 °C (from dichloromethane:light petroleum ether). The NMR data had minor traces of impurities, so for NMR, the sample was purified by chromatography and eluted with DCM. λ_max_ (EtOH)/nm 224 (log ε 3.7), 282 (3.4), and 438 (3.4); ν_max_ (diamond)(cm^–1^) 2987w, 1634s, 1579s, 1532s,1489s, 1367s, 1323s, 1263s, 1078s, 1046s, 1023s, 922s, 870s, 806s and 733s; δ_H_ (400 MHz; D_7_DMF) 1.58 (3H, t, *J* = 8.0), 3.46 (1H, s), 4.47 (2H, q, *J* = 8.0), 6.74 (1H, s), 6.98 (1H, dd, *J* = 8.0 and 2.0), 7.06 (1H, dt, *J* = 8.0 and 2.0), 7.09–7.17 (2H, m) and 7.50 (1H, s); δ_C_ (100.1 MHz; CDCl_3_) 14.3, 31.6, 65.9, 98.5, 111.4, 113.4, 115.4, 123.2, 124.6, 129.6, 132.1, 137.2, 141.7, 144.5 and 152.7; *m*/*z* (Orbitrap ASAP) 287.1027 (M^+^ + H, 100%) C_15_H_14_N_2_O_4_H requires 287.1032.
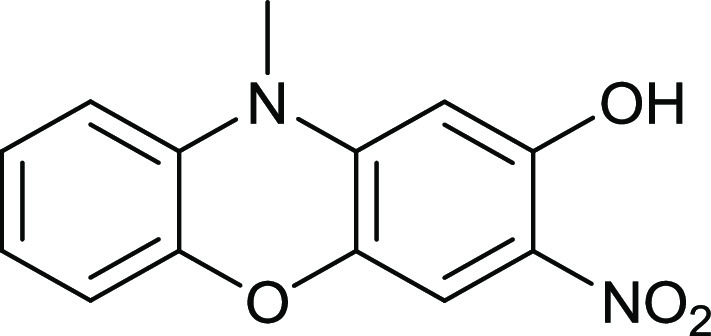


### 2-Hydroxy-3-nitro-*N*-methylphenoxazine **17**

*N*-Methyl-3,4-dinitrophenoxazine (50 mg, 0.17 mmol) was mixed with DMSO (5 mL) and water (1 mL of water with 20 mg of KOH) and then heated at 70 °C for 1.5 h with stirring. The mixture was diluted with water (200 mL), extracted with DCM (50 mL), backwashed with water (100 mL), dried over MgSO_4_, and purified by chromatography. Elution with DCM gave the *title compound* (12 mg, 27%) as red crystals; mp 210–211 °C (from dichloromethane:light petroleum ether). λ_max_ (EtOH)/nm 226 (log ε 4.4), 285(3.6) and 475(3.8); ν_max_ (diamond)(cm^–1^) 2926w,1640w, 1607w, 1574w, 1488s, 1419s, 1398s, 1349s, 1242s, 1197s, 1116s, 1084s, 1039s, 855s, 811s, 742s, 698s, 677s, 652s, 458s and 438s; δ_H_ (400 MHz; D_7_DMF) 3.48 (3H, s), 6.62 (1H, s), 7.02 (1H, d, *J* = 8.0), 7.10 (1H, td, *J* = 8.0 and 4.0), 7.15–7.22 (2H, m), 7.42 (1H, s) and 11.48–11.41 (1H, s, br); δ_C_ (100.1 MHz; D_7_DMF) 31.9, 100.1, 108.5, 114.2, 115.7, 123.8, 124.5, 125.4, 130.8, 138.1, 144.0, 144.3 and 155.4; *m*/*z* (Orbitrap ASAP) 259.0716 (M^+^ + H, 100%) C_13_H_10_N_2_O_4_H requires 259.0719.
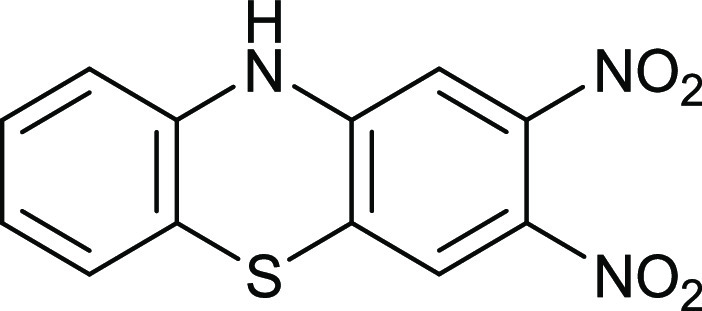


### 2,3-Dinitrophenothiazine **19**

4,5-Difluoro-1,2-dinitrobenzene **9** (110 mg, 0.539 mmol) in EtOH (30 mL) was mixed with 2-aminothiophenol **15** (68 mg, 0.54 mmol) and Na_2_CO_3_ (500 mg) and then stirred at 70 °C for 20 h. After cooling, the mixture was added to water (200 mL), left standing for 1 h, filtered through a sinter grade No. 4, and air-dried to give the *title compound* (126 mg, 81%) as dark red crystals, mp 226–227 °C (from dichloromethane:light petroleum ether). λ_max_ (EtOH)/nm 233 (log ε 4.1), 296 (3.9) and 476 (3.3); ν_max_ (diamond)(cm^–1^) 3327s, 1600s, 1568s, 1527s, 1568s, 1527s, 1500s, 1474s, 1354s, 1295s, 1264s, 1230s, 1150s, 1106s, 883s, 847s, 821s and 753s; δ_H_ (400 MHz; CDCl_3_) 6.93 (1H, d, *J* = 8.0), 7.08–7.17 (2H, m), 7.25 (1H, d, *J* = 8.0), 7.28 (1H, s), 7.97 (1H, s) and 10.04 (1H, NH, br); δ_C_ (100.1 MHz; D_7_DMF) 108.9, 115.2, 116.4, 122.1, 123.6, 124.9, 126.7, 128.9, 134.4, 138.5, 144.3 and 147.8; *m*/*z* (Orbitrap ASAP) 290.0243 (M^+^ + H, 100%) C_12_H_7_N_3_O_4_SH requires 290.0236.
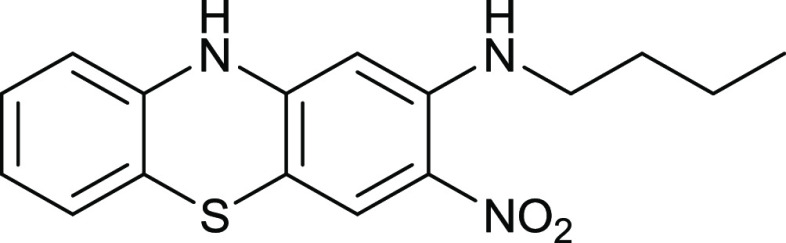


### 2-Butylamino-3-nitrophenothiazine **20**

3,4-Dinitrophenothiazine **19** (80 mg, 0.27 mmol) in EtOH (30 mL) was mixed with butylamine (61 mg, 0.69 mmol) and heated at 70 °C for 48 h. After cooling, the mixture was mixed with water (170 mL) and filtered, and the precipitate was purified by chromatography. Elution with DCM gave the *title compound* (39 mg, 46%) as dark red crystals, mp 242–243 °C (from dichloromethane:light petroleum ether). λ_max_ (EtOH)/nm 261(log ε 4.3), 306(3.8) 403sh(3.5) and 481(3.8); ν_max_ (diamond)(cm^–1^) 3280w, 3199w, 3147w, 2952w, 2928w, 2861w, 1616s, 1591s, 1553s, 1464s, 1451s, 1418s, 1406s, 1330s, 1298s, 1262s, 1231s, 1169s, 1067s, 877s, 832s, 729s, 674s, 532s and 489s; δ_H_ (400 MHz; CDCl_3_) 1.13 (3H, t, *J* = 8.0), 1.60 (2H, s, *J* = 8.0), 1.84 (2H, q, *J* = 8.0), 3.49 (2H, q, *J* = 8.0), 6.41 (1H, s), 7.02 (1H, d, *J* = 8.0), 7.07 (1H, t, *J* = 8.0), 7.21 (1H, d, *J* = 8.0), 7.25 (1H, t, *J* = 8.0), 7.86 (1H, s), 8.63 (1H, s, br, NH) and 9.91 (1H, s, NH); δ_C_ (100.1 MHz; D_7_DMF) 13.5, 20.1, 30.8, 42.5, 95.4, 106.0, 115.9, 116.9, 123.1, 123.3, 126.2, 126.3, 127.8, 138.3, 147.6, and 148.7 (one aliphatic peak is missing); *m*/*z* (Orbitrap ASAP) 316.1118 (M^+^ + H, 100%) C_16_H_17_N_3_O_2_SH requires 316.1120.

## Crystal Structures

The crystal structures of compounds **13**–**17**, **19**, and **20** were established using intensity data collected on a Rigaku CCD diffractometer at 100 K. The structures were routinely solved by dual-space methods using SHELXT,^47^ and the structural models were completed and optimized by refinement against |*F*|^2^ with SHELXL-2019.^48^ The O- and N-bound hydrogen atoms (if any) were located in difference maps, and their positions were freely refined, except for compound **20**, where they were geometrically placed. The C-bound hydrogen atoms were placed in idealized locations (C–H = 0.95–0.99 Å) and were refined as riding atoms. The methyl groups (if any) were allowed to rotate, but not to tip, to best fit the electron density. The constraint *U*_iso_(H) = 1.2*U*_eq_(carrier) or 1.5*U*_eq_(methyl carrier) was applied in all cases. The data quality for compound **20** is poor, but the structure has been unambiguously established. Full details of the structures and refinements are available in the deposited cifs.

Crystal data for compound **13** C_12_H_7_N_3_O_5_, red plate 0.23 × 0.14 × 0.03 mm^3^, *M*_r_ = 273.21, orthorhombic, space group *P*2_1_2_1_2_1_ (No. 19), *a* = 6.6145 (4) Å, *b* = 11.2990 (6) Å, *c* = 14.7681 (9) Å, *V* = 1103.73 (11) Å^3^, *Z* = 4, *T* = 100 K, Cu Kα radiation, λ = 1.54178 Å, μ = 1.132 mm^–1^, ρ_calc_ = 1.644 g cm^–3^, 5772 reflections measured (9.9 ≤ 2θ ≤ 140.1°), 2001 unique (*R*_Int_ = 0.073), *R*(*F*) = 0.064 [1722 reflections with *I* > 2σ(*I*)], *w*R**(*F*^2^) = 0.177 (all data), Δρ_min,max_ (*e* Å^–3^) = −0.33, + 0.45, Flack absolute structure parameter 0.0 (4), CCDC deposition number 2291273.

Crystal data for compound **14** C_13_H_9_N_3_O_5_, orange lath 0.30 × 0.08 × 0.02 mm^3^, *M*_r_ = 287.23, orthorhombic, space group *P*2_1_2_1_2_1_ (No. 19), *a* = 6.73506 (4) Å, *b* = 10.46527 (8) Å, *c* = 16.90115 (15) Å, *V* = 1191.263 (16) Å^3^, *Z* = 4, *T* = 100 K, Cu Kα radiation, λ = 1.54178 Å, μ = 1.079 mm^–1^, ρ_calc_ = 1.602 g cm^–3^, 35898 reflections measured (9.9 ≤ 2θ ≤ 153.8°), 2411 unique (*R*_Int_ = 0.067), *R*(*F*) = 0.030 [2384 reflections with *I* > 2σ(*I*)], *w*R**(*F*^2^) = 0.086 (all data), Δρ_min,max_ (*e* Å^–3^) = −0.23, + 0.15, Flack absolute structure parameter 0.09 (7), CCDC deposition number 2291274.

Crystal data for compound **15** C_17_H_19_N_3_O_3_, orange lath 0.08 × 0.05 × 0.03 mm^3^, *M*_r_ = 313.35, triclinic, space group *P*1̅ (No. 2), *a* = 7.2750 (3) Å, *b* = 12.8080 (5) Å, *c* = 16.1101 (6) Å, α = 78.021 (3)°, β = 87.636 (3)°, γ = 85.439 (3)°, *V* = 1463.30 (10) Å^3^, *Z* = 4, *T* = 100 K, synchrotron radiation, λ = 0.6889 Å, μ = 0.093 mm^–1^, ρ_calc_ = 1.422 g cm^–3^, 16071 reflections measured (3.2 ≤ 2θ ≤ 47.0°), 4712 unique (*R*_Int_ = 0.169), *R*(*F*) = 0.090 [2866 reflections with *I* > 2σ(*I*)], *w*R**(*F*^2^) = 0.259 (all data), Δρ_min,max_ (*e* Å^–3^) = −0.37, + 0.32, CCDC deposition number 2291275.

Crystal data for compound **16** C_15_H_14_N_2_O_4_, orange slab 0.32 × 0.19 × 0.05 mm^3^, *M*_r_ = 286.28, monoclinic, space group *P*2_1_/*n* (No. 14), *a* = 13.67695 (14) Å, *b* = 13.86021 (15) Å, *c* = 13.70349 (13) Å, β = 98.2282 (10)°, *V* = 2570.97 (5) Å^3^, *Z* = 8, *T* = 100 K, Cu Kα radiation, λ = 1.54178 Å, μ = 0.909 mm^–1^, ρ_calc_ = 1.479 g cm^–3^, 74062 reflections measured (8.5 ≤ 2θ ≤ 149.5°), 5214 unique (*R*_Int_ = 0.022), *R*(*F*) = 0.033 [4809 reflections with *I* > 2σ(*I*)], *w*R**(*F*^2^) = 0.100 (all data), Δρ_min,max_ (*e* Å^–3^) = −0.26, + 0.29, CCDC deposition number 2291276.

Crystal data for compound **17** C_13_H_10_N_2_O_4_, red plate 0.12 × 0.05 × 0.02 mm^3^, *M*_r_ = 258.23, triclinic, space group *P*1̅ (No. 2), *a* = 7.8187 (8) Å, *b* = 8.3024 (9) Å, *c* = 9.1432 (8) Å, α = 79.273 (8)°, β = 81.368 (8)°, γ = 67.723 (10)°, *V* = 537.56 (10) Å^3^, *Z* = 2, *T* = 100 K, Cu Kα radiation, λ = 1.54178 Å, μ = 1.018 mm^–1^, ρ_calc_ = 1.595 g cm^–3^, 9116 reflections measured (11.6 ≤ 2θ ≤ 149.7°), 2139 unique (*R*_Int_ = 0.038), *R*(*F*) = 0.060 [1702 reflections with *I* > 2σ(*I*)], *w*R**(*F*^2^) = 0.186 (all data), Δρ_min,max_ (*e* Å^–3^) = −0.38, + 0.38, CCDC deposition number 2291277.

Crystal data for compound **19** C_12_H_7_N_3_O_4_S, black prism 0.59 × 0.19 × 0.11 mm^3^, *M*_r_ = 289.27, orthorhombic, space group *P*2_1_2_1_2_1_ (No. 19), *a* = 6.83300 (12) Å, *b* = 11.01073 (17) Å, *c* = 15.6038 (2) Å, *V* = 1173.97 (3) Å^3^, *Z* = 4, *T* = 100 K, Mo Kα radiation, λ = 0.71073 Å, μ = 0.294 mm^–1^, ρ_calc_ = 1.637 g cm^–3^, 38956 reflections measured (4.5 ≤ 2θ ≤ 66.4°), 4233 unique (*R*_Int_ = 0.055), *R*(*F*) = 0.030 [4038 reflections with *I* > 2σ(*I*)], *w*R**(*F*^2^) = 0.076 (all data), Δρ_min,max_ (*e* Å^–3^) = −0.24, + 0.40, Flack absolute structure parameter 0.40 (6), CCDC deposition number 2291278.

Crystal data for compound **20** 2(C_16_H_17_N_3_O_2_S)·CH_2_Cl_2_, orange plate 0.39 × 0.31 × 0.04 mm^3^, *M*_r_ = 715.70, monoclinic, space group *P*2_1_/*c* (No. 14), *a* = 7.4727 (2) Å, *b* = 15.1810 (5) Å, *c* = 29.2495 (10) Å, β = 94.148 (3)°, *V* = 3309.46 (18) Å^3^, *Z* = 4, *T* = 100 K, Cu Kα radiation, λ = 1.54178 Å, μ = 3.344 mm^–1^, ρ_calc_ = 1.436 g cm^–3^, 30254 reflections measured (6.1 ≤ 2θ ≤ 147.7°), 6518 unique (*R*_Int_ = 0.073), *R*(*F*) = 0.117 [5139 reflections with *I* > 2σ(*I*)], *w*R**(*F*^2^) = 0.326 (all data), Δρ_min,max_ (*e* Å^–3^) = −0.88, + 1.49, CCDC deposition number 2291279.

## Supporting Information Available

The proton and carbon NMR data for all compounds in the Experimental Section (Figures S1–S19) (PDF)

## Accession Codes

CCDC 2291273 (compound **13**), CCDC 2291274 (compound **14**), CCDC 2291275 (compound **15**), CCDC 2291276 (compound **16**), CCDC 2291277 (compound **17**), CCDC 2291278 (compound **19**), and CCDC 2291279 (compound **20**) contain the supplementary crystallographic data for this paper. These data can be obtained free of charge from the Cambridge Crystallographic Data Centre via www.ccdc.cam.ac.uk/data request/cif.
