# Supplementary material for: New Functionalized Phenoxazines and Phenothiazines
Source: ACS Omega. 2023 Nov 9;8(46):44163–71. doi: 10.1021/acsomega.3c06461 (PMC10666145; doi:10.1021/acsomega.3c06461)

Supplementary Information SI

New functionalised phenoxazines and phenothiazines

M. John Plater* and William T. A. Harrison

Department of Chemistry, University of Aberdeen, Meston Walk, Aberdeen, AB24 3UE

m.j.plater@abdn.ac.uk

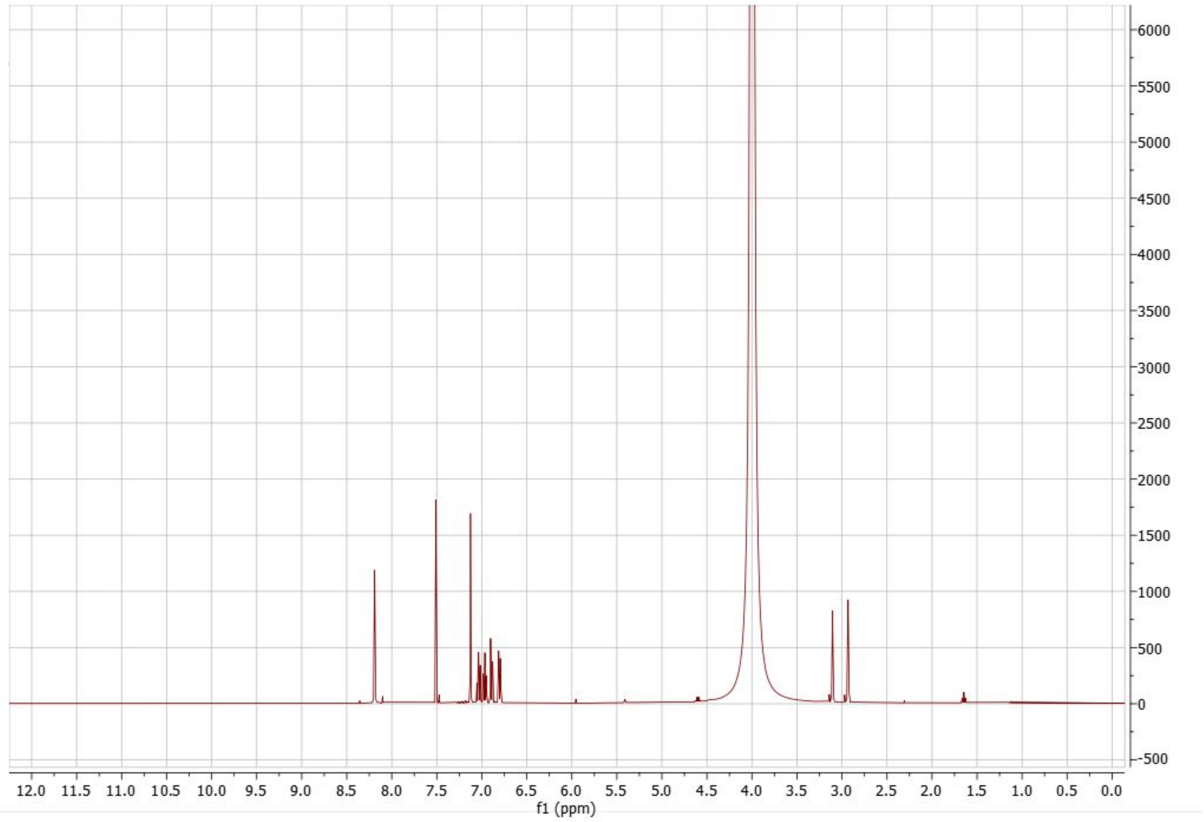

Figure S1 13 400 MHz NMR (D_7DMF) Proton

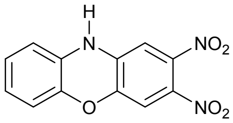

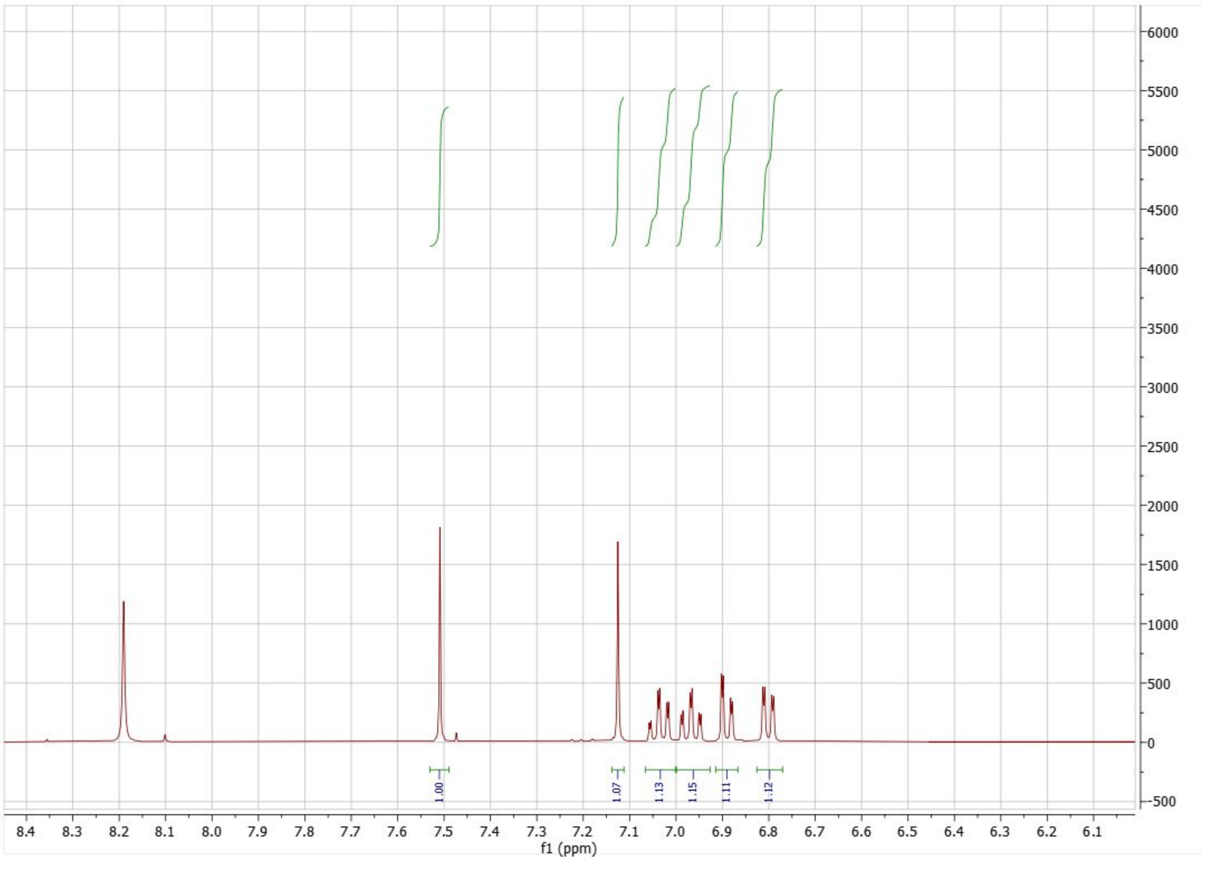

Figure S2 13 400 MHz NMR (D_7DMF) Proton Expansion

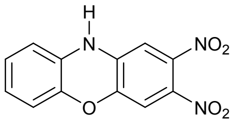

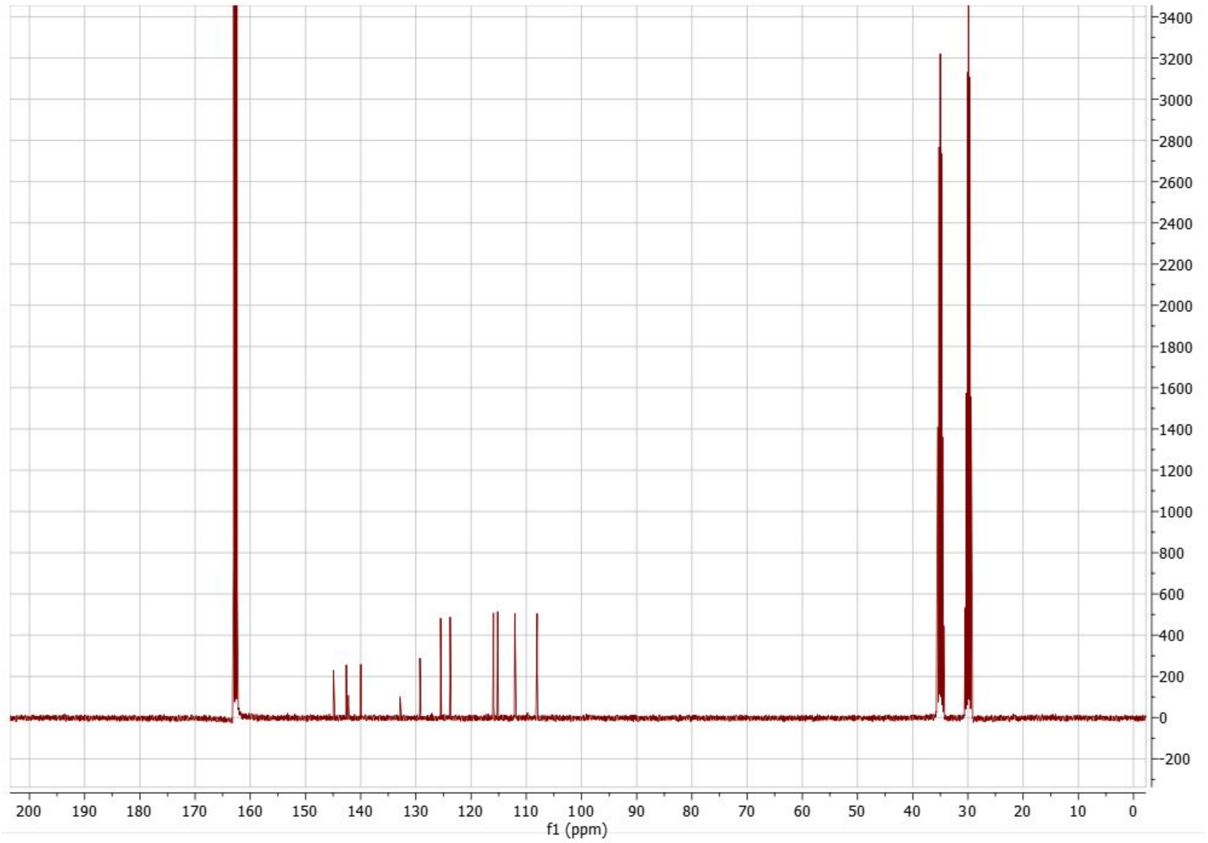

Figure S3 13 400 MHz NMR (D_7DMF) Carbon

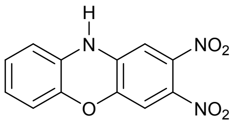

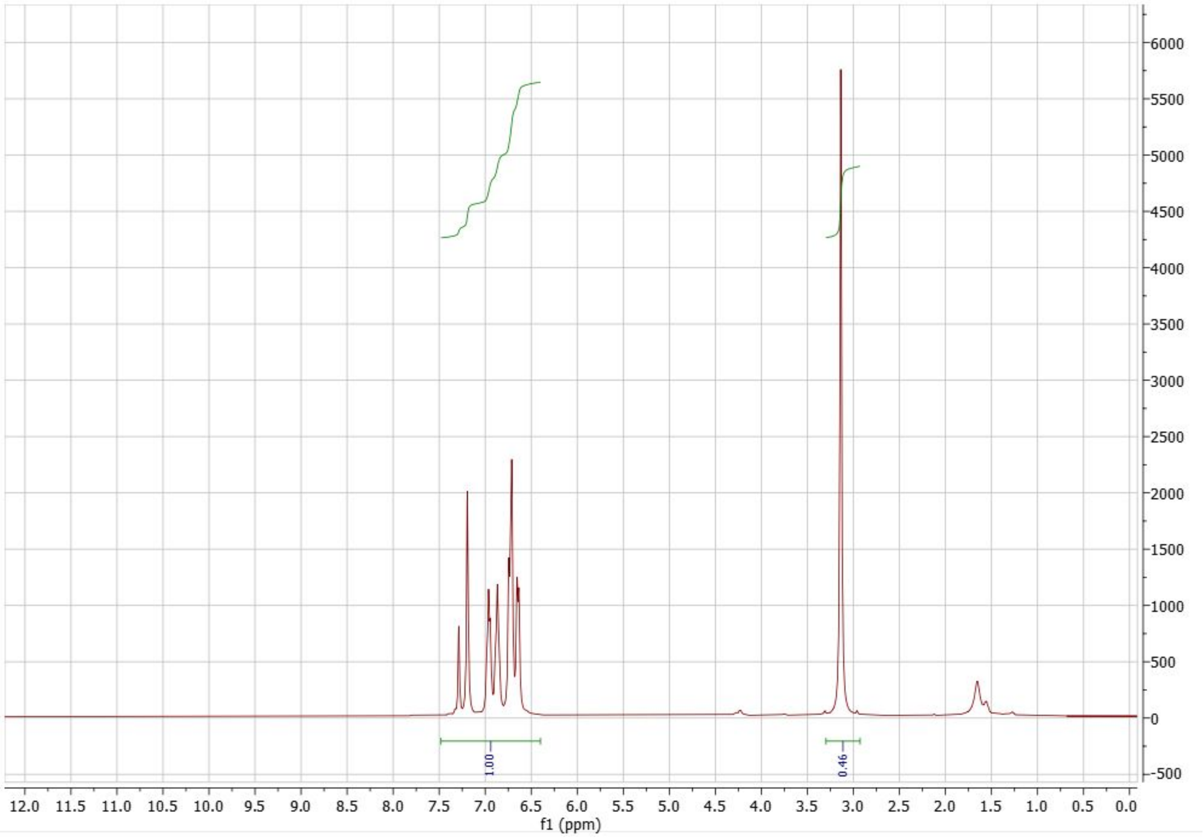

Figure S4 14 400 MHz NMR ($CDCl_3$) Proton

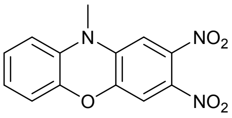

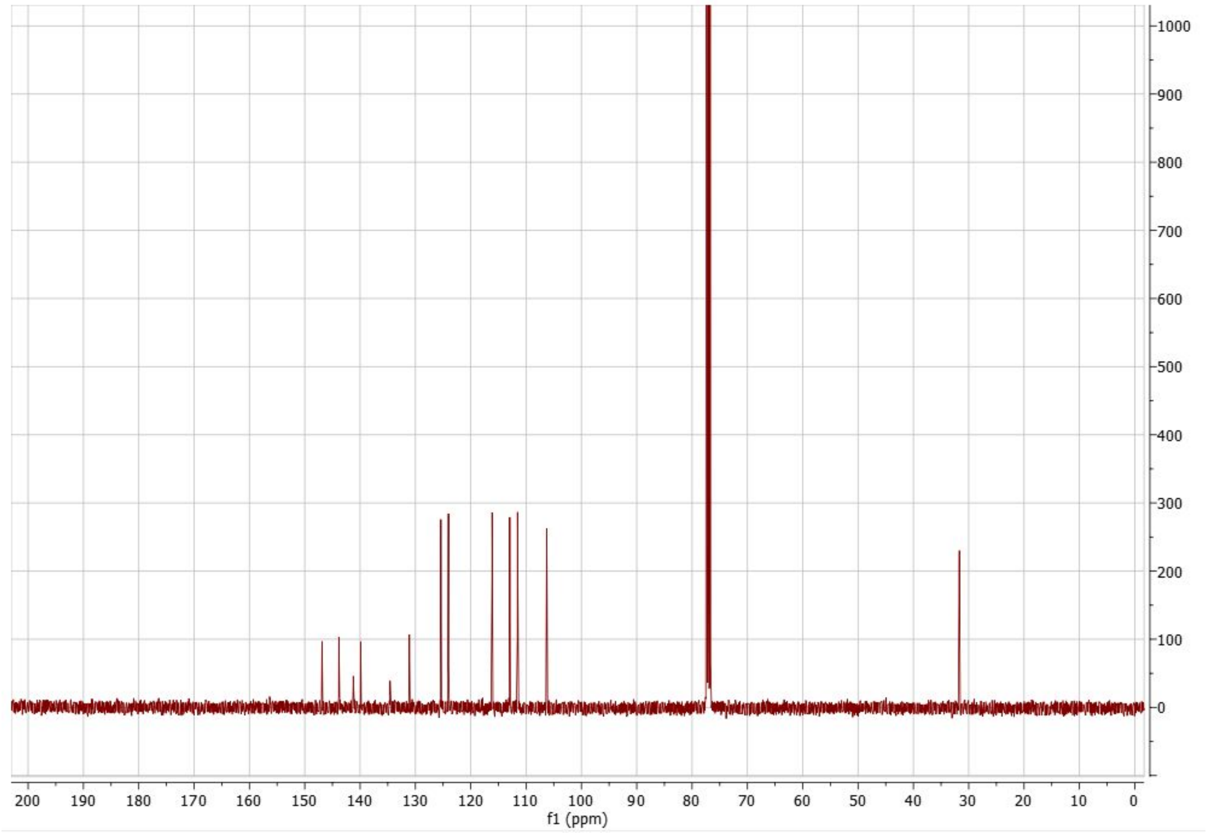

Figure S5 14 400 MHz NMR ($CDCl_3$) Carbon

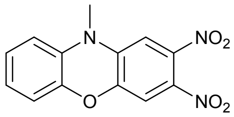

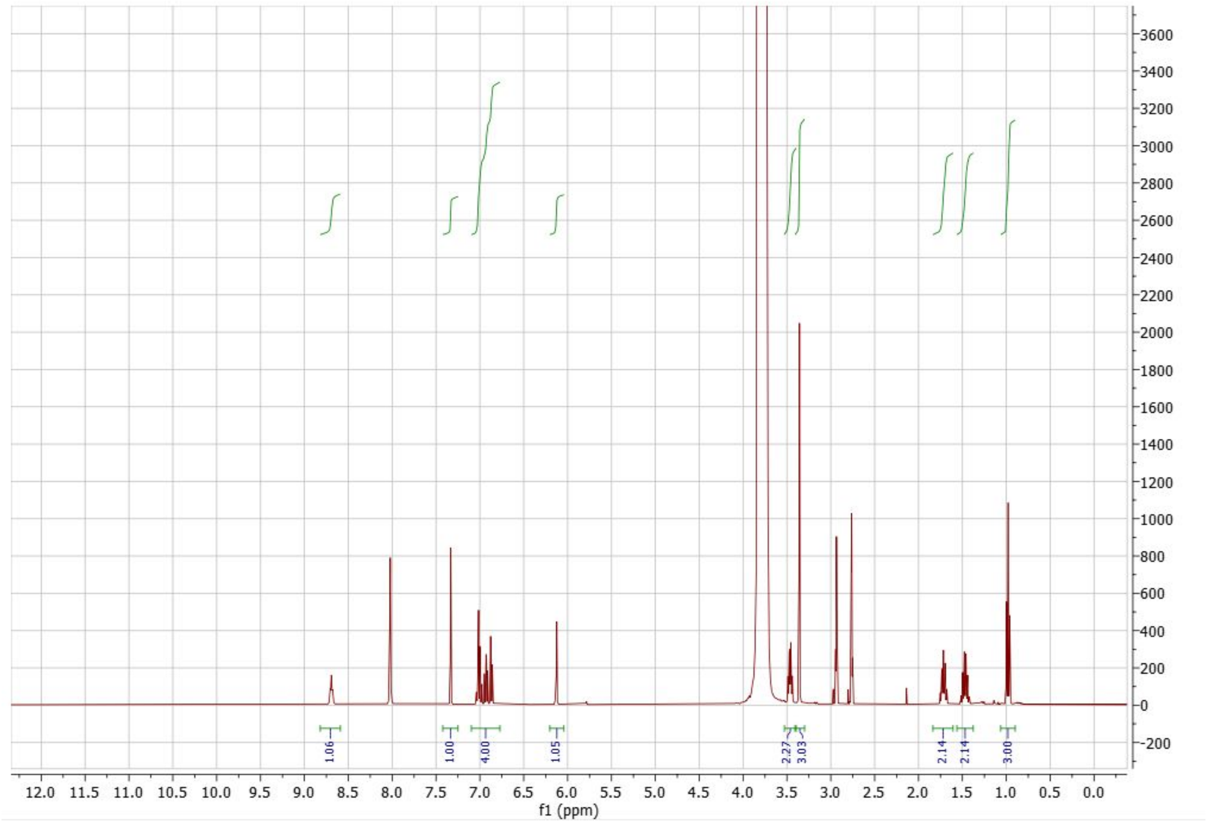

Figure S6 15 400 MHz NMR (D_7DMF) Proton

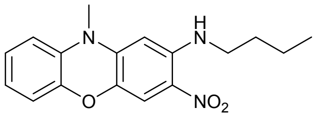

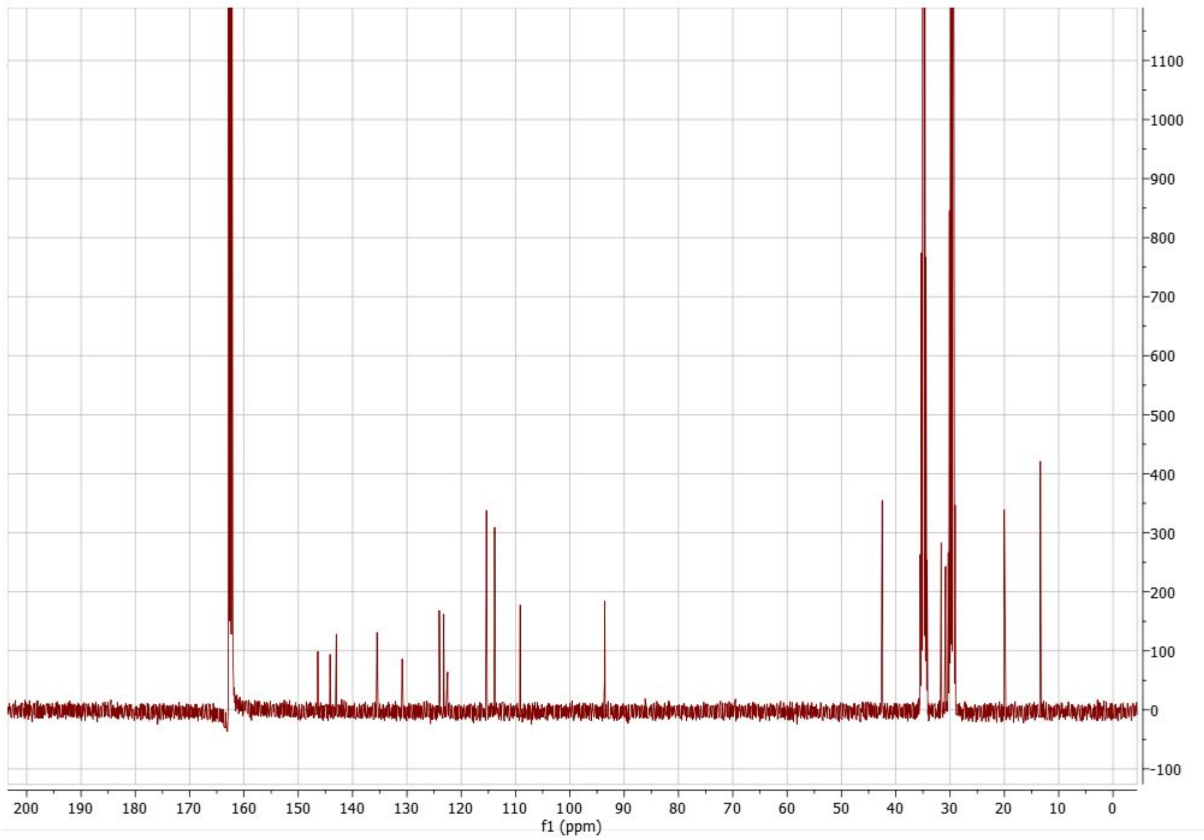

Figure S7 15 400 MHz NMR (D_7DMF) Carbon

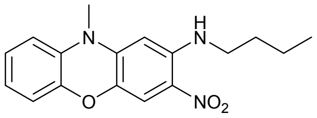

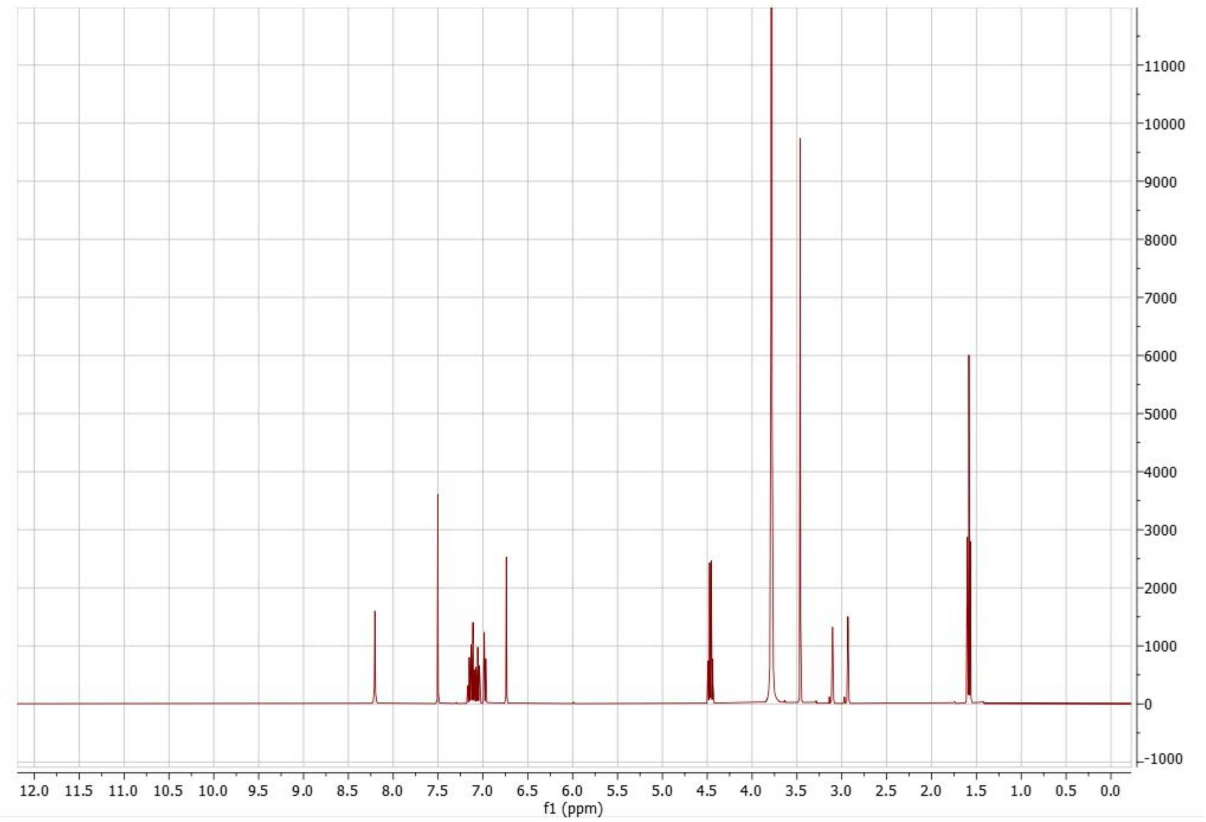

Figure S8 16 400 MHz NMR (D_7DMF) Proton

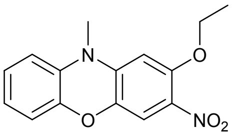

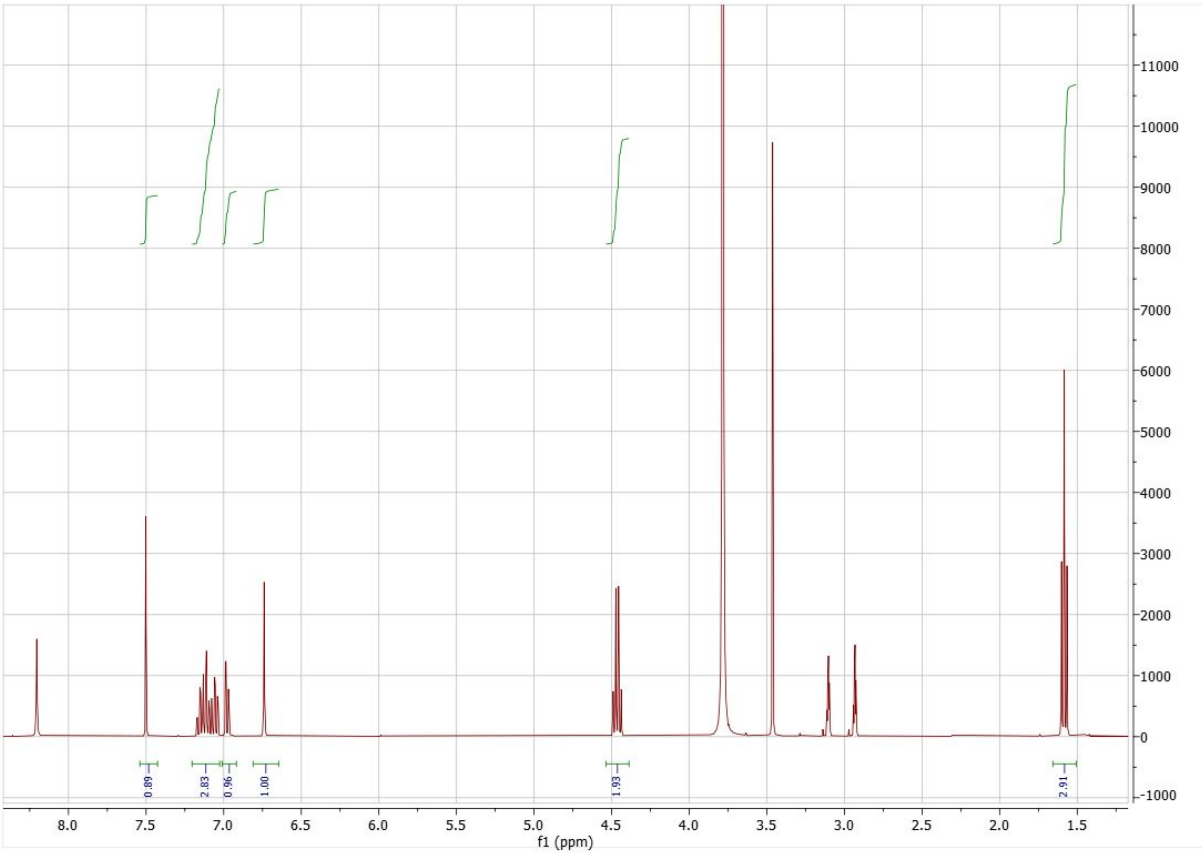

Figure S9 16 400 MHz NMR (D_7DMF) Proton Expansion

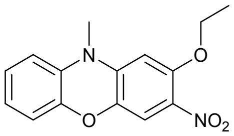

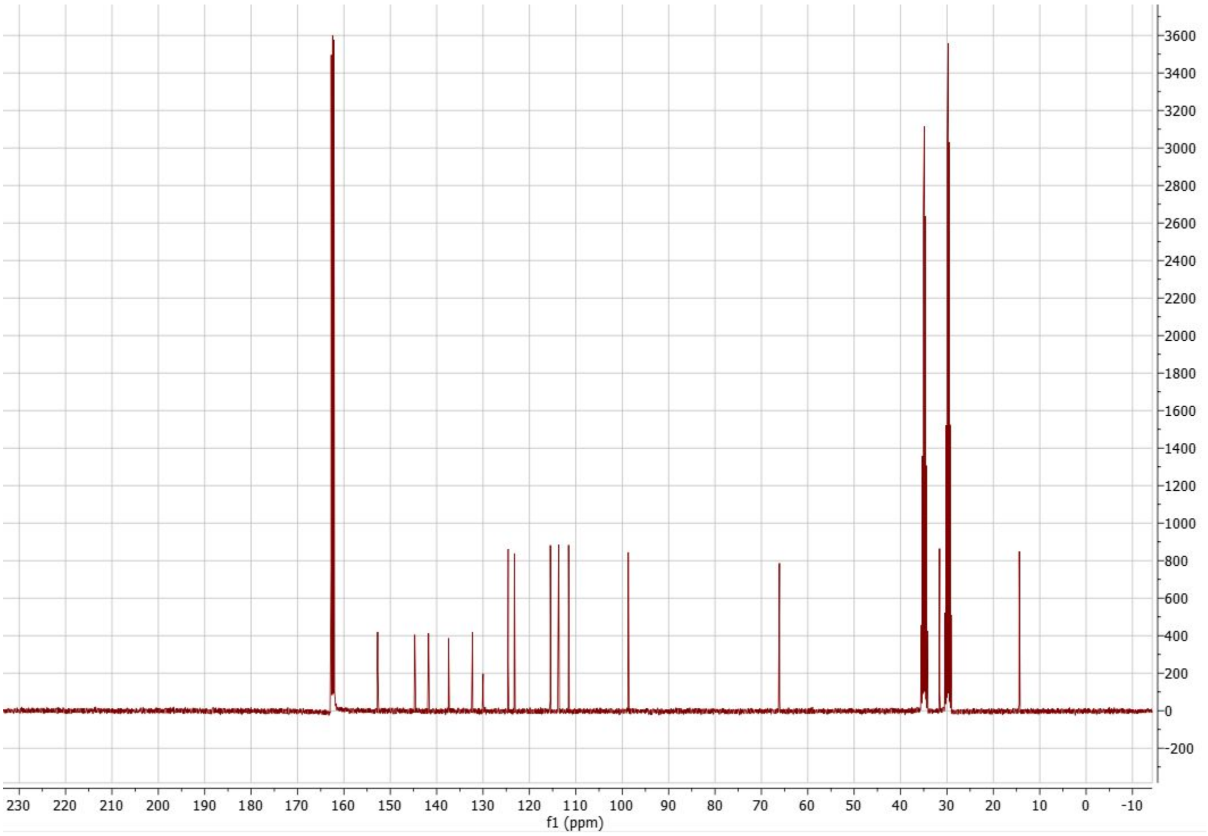

Figure S10 16 400 MHz NMR (D_7DMF) Carbon

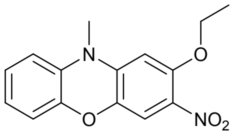

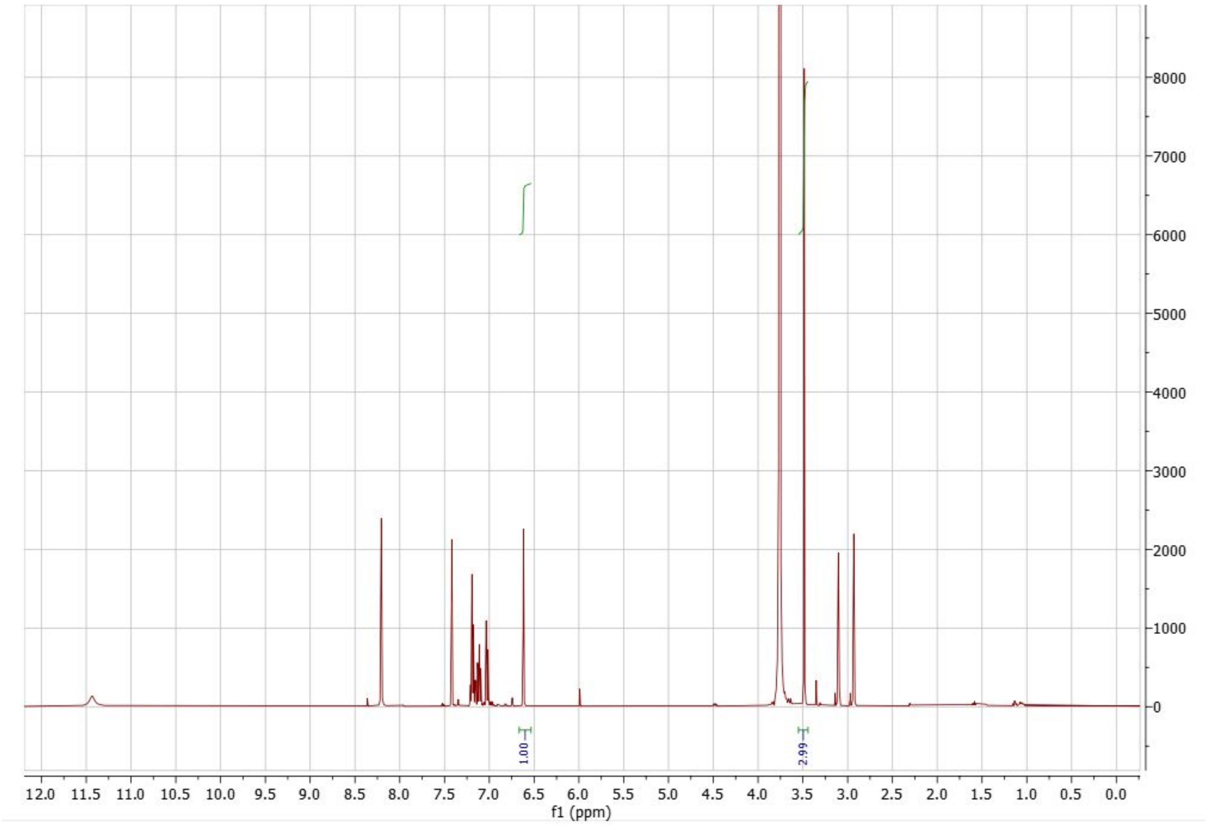

Figure S11 17 400 MHz NMR (D_7DMF) Proton

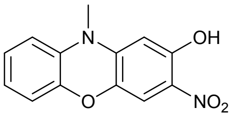

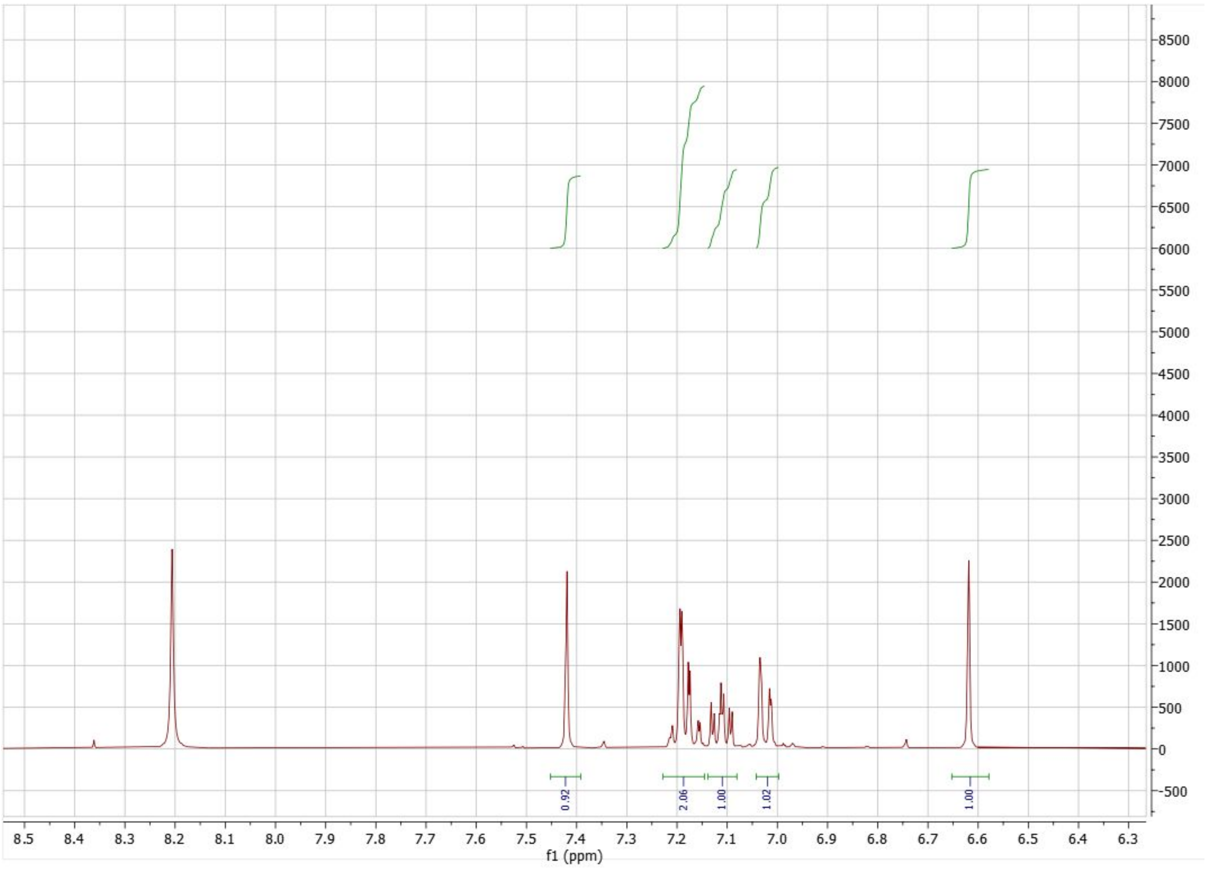

Figure S12 17 400 MHz NMR (D_7DMF) Proton (Expansion)

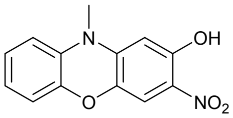

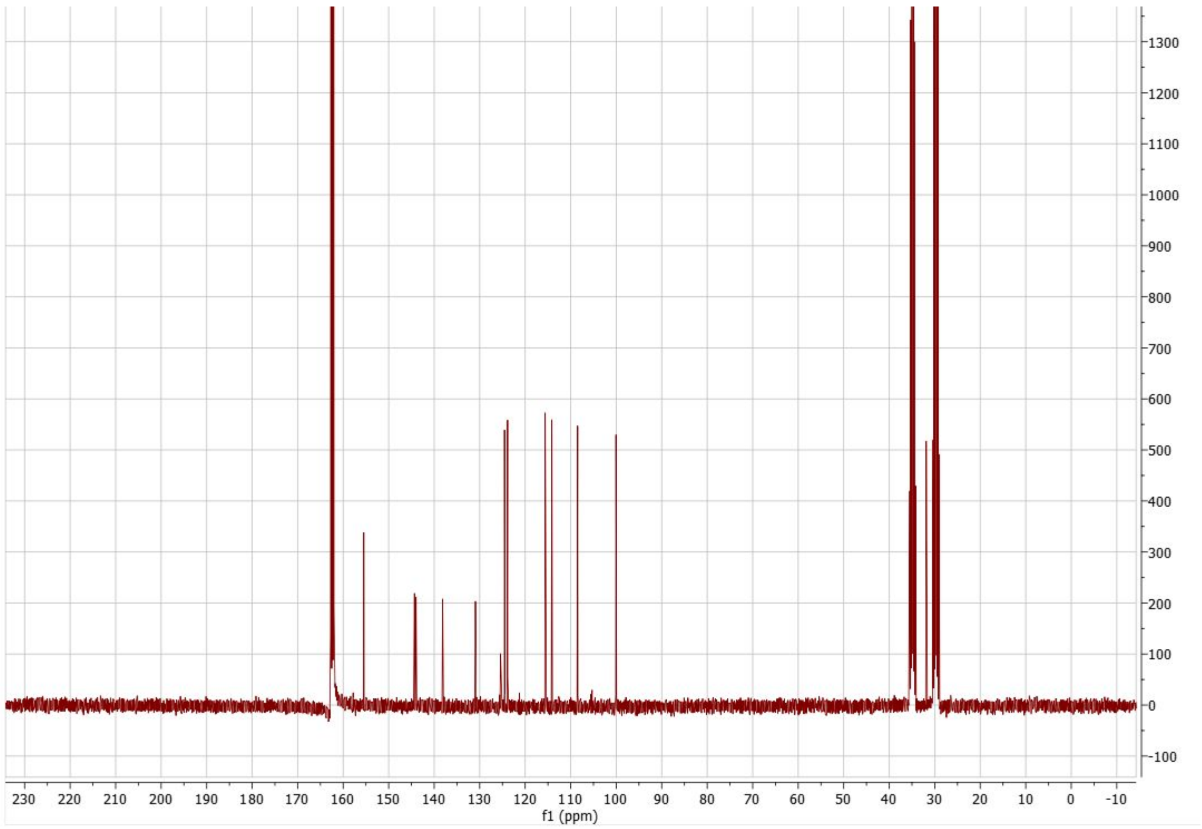

Figure S13 13 400 MHz NMR (D_7DMF) Carbon

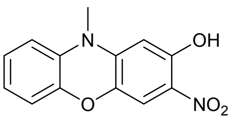

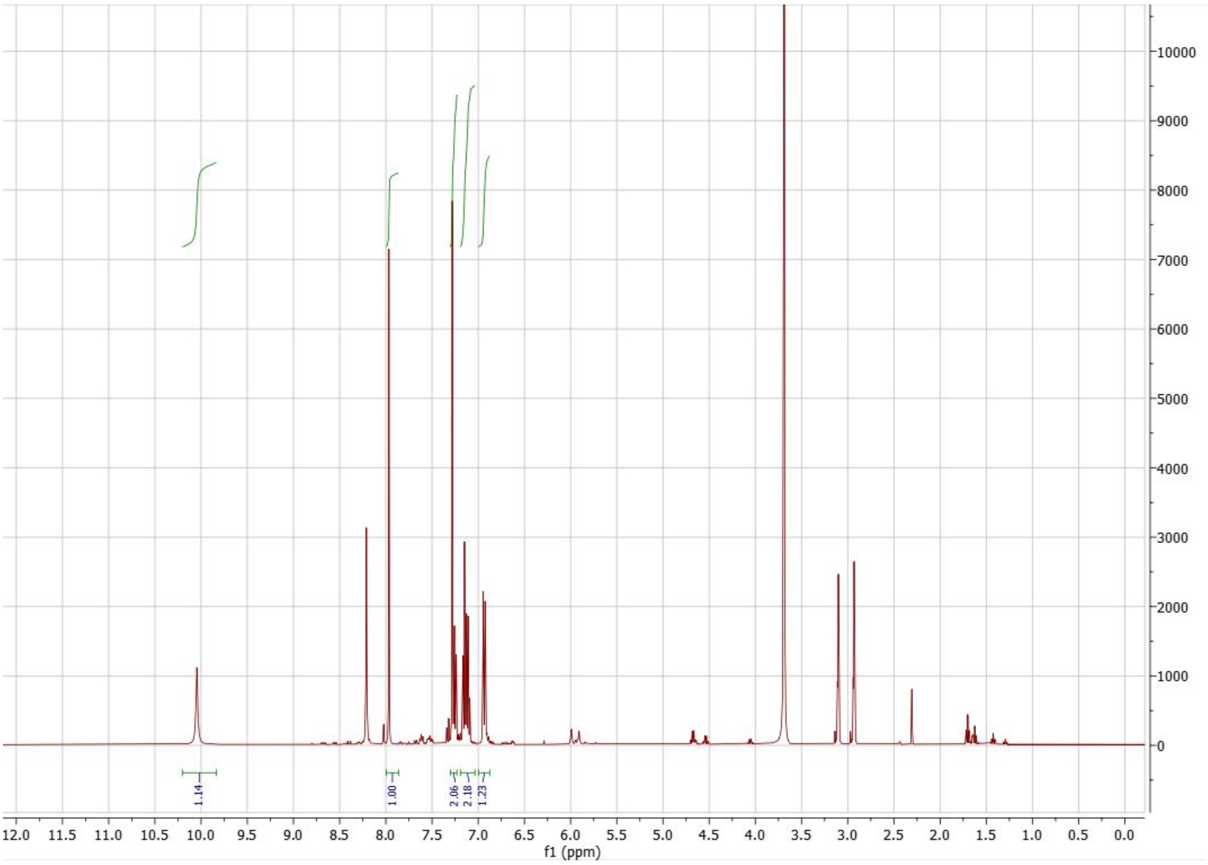

Figure S14 19 400 MHz NMR (D_7DMF) Proton

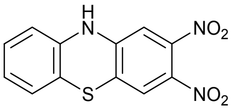

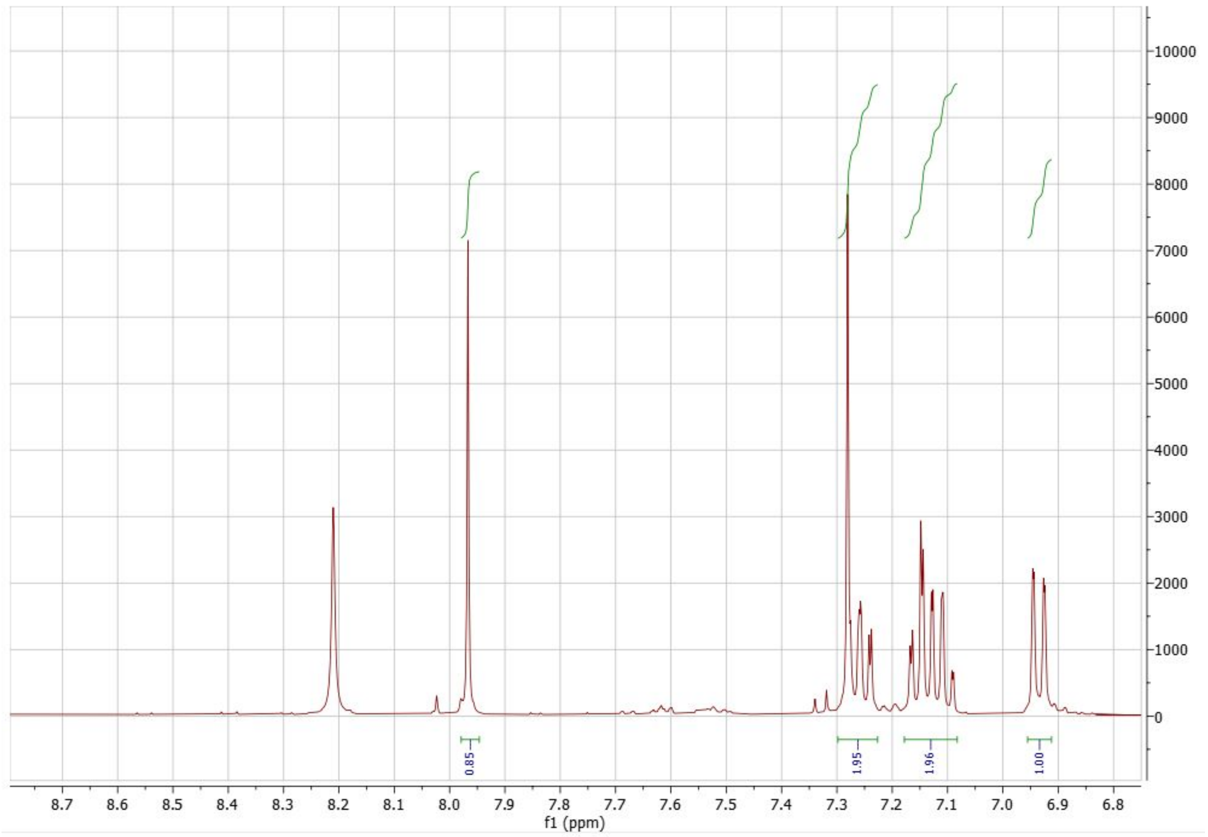

Figure S15 19 400 MHz NMR (D_7DMF) Proton Expansion

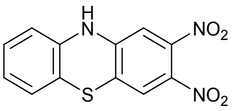

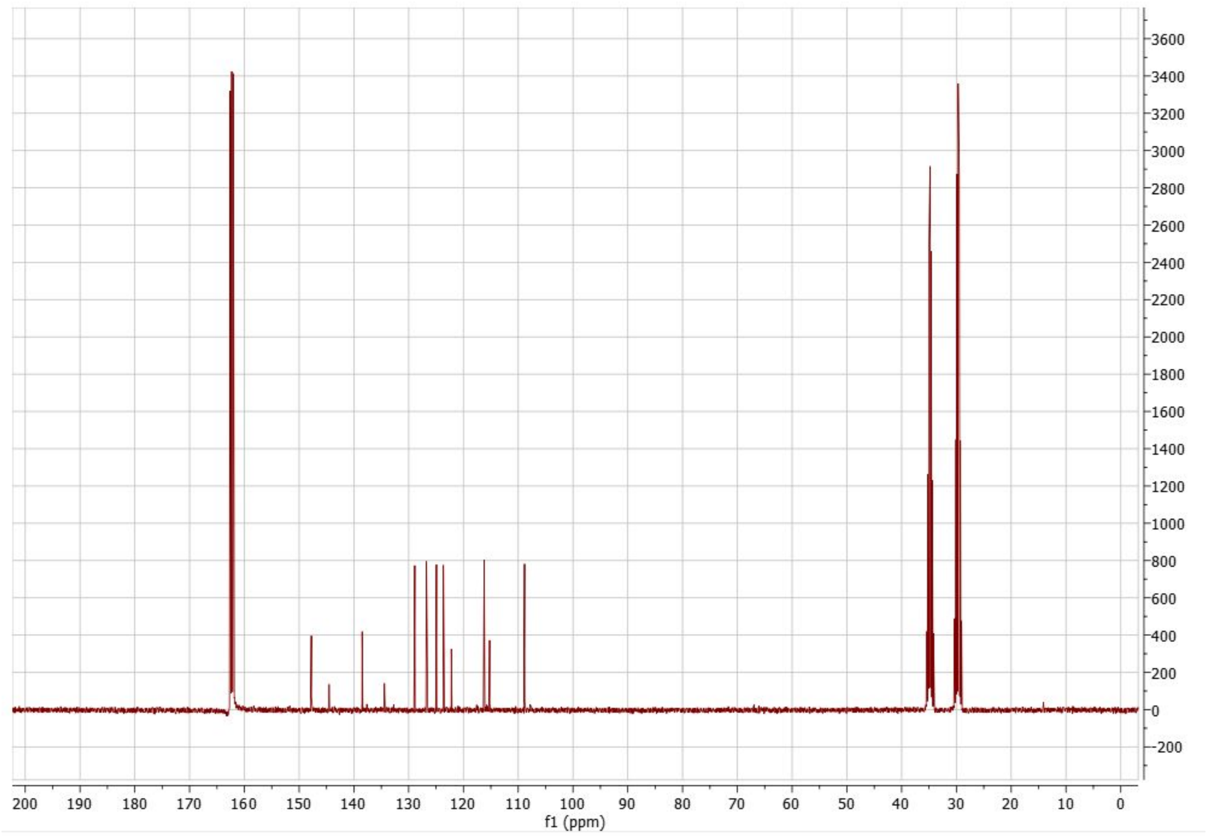

Figure S16 19 400 MHz NMR (D_7DMF) Carbon

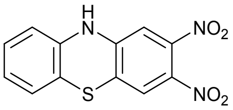

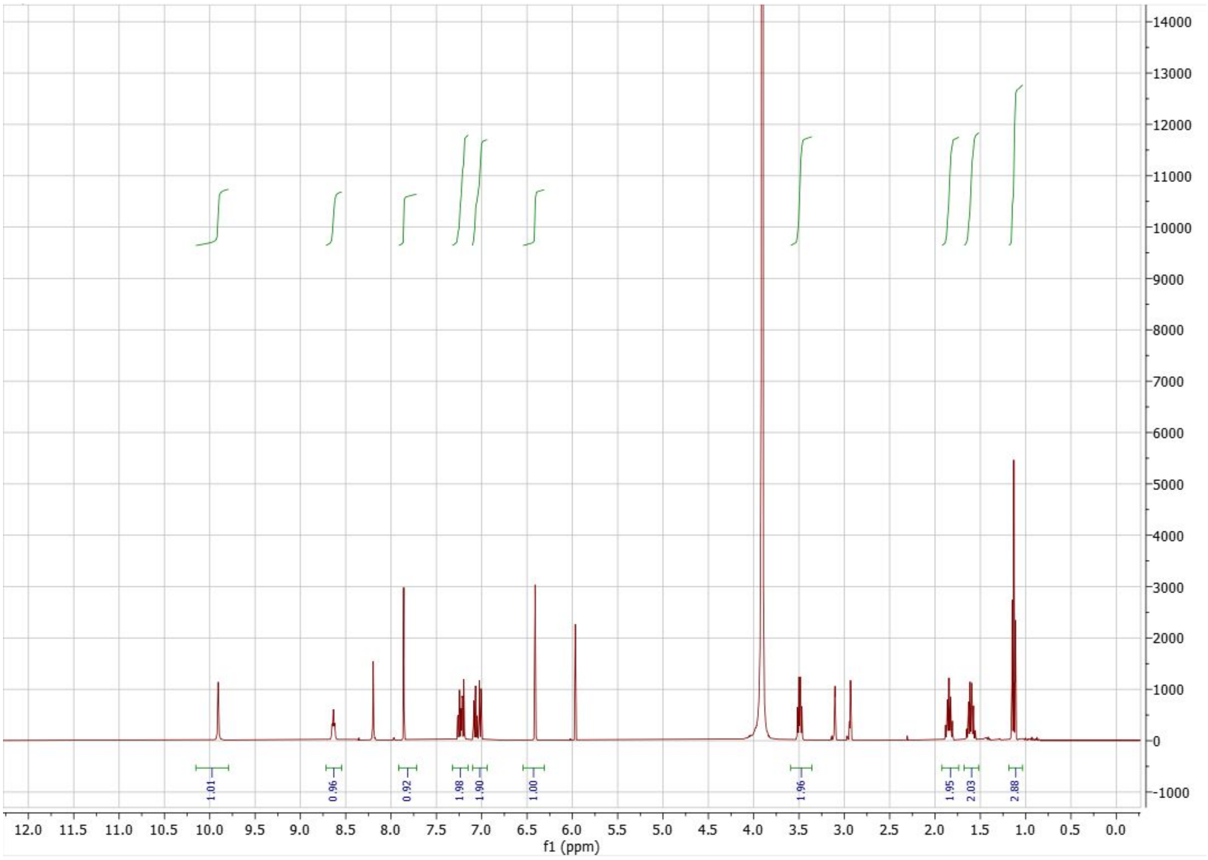

Figure S17 20 400 MHz NMR (D_7DMF) Proton

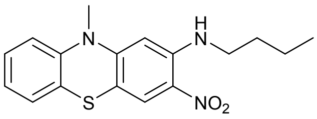

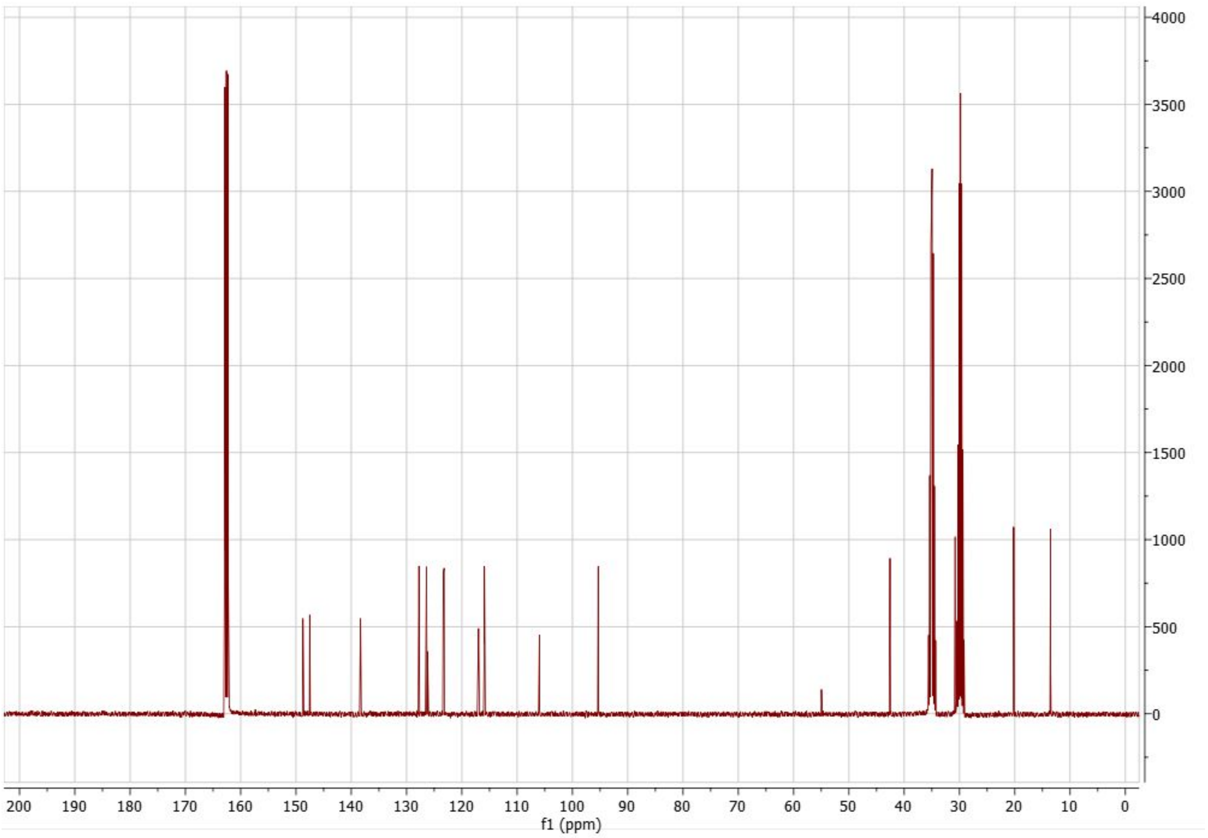

Figure S18 20 400 MHz NMR (D_7DMF) Carbon

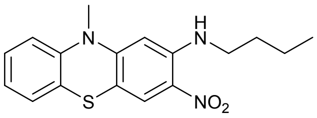

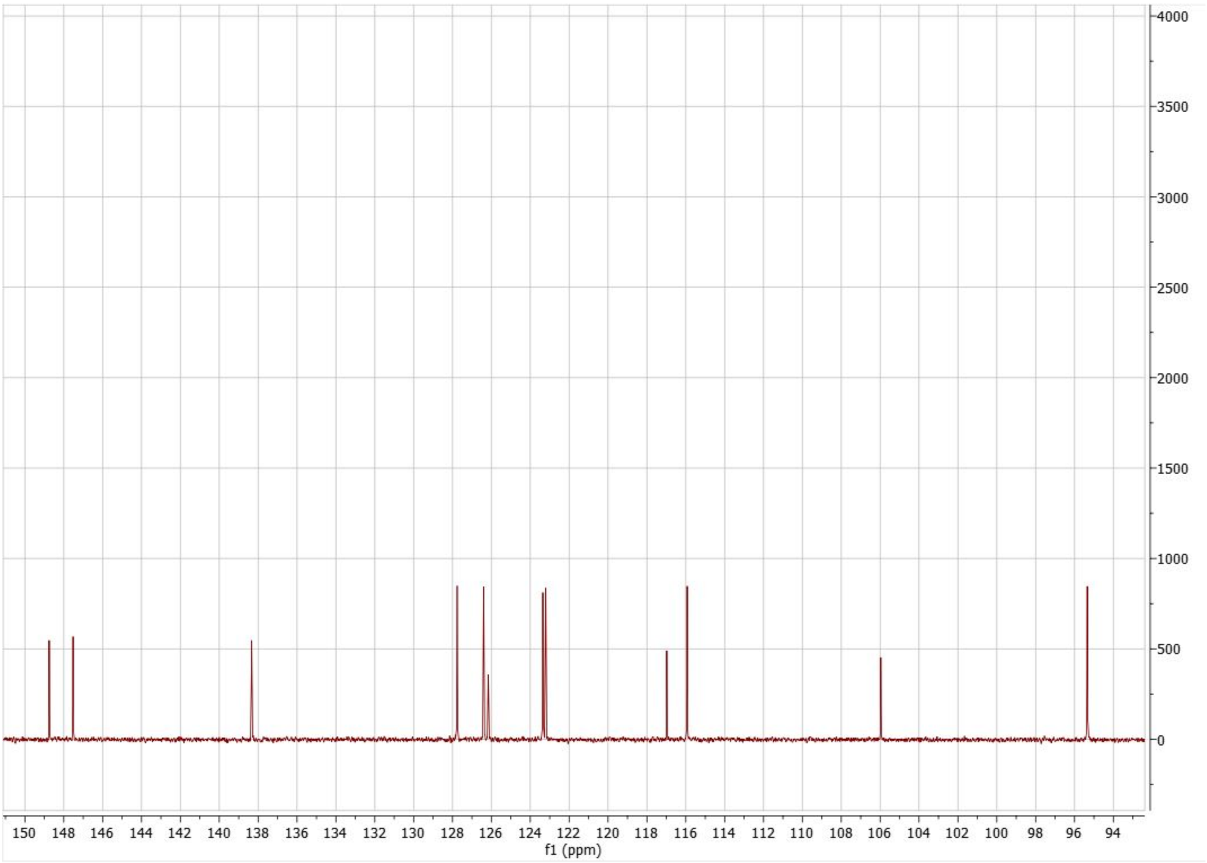

Figure S19 20 400 MHz NMR (D_7DMF) Carbon Expansion

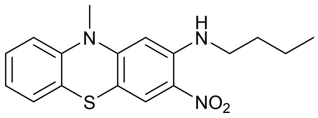

Supplement: Supplementary file 1 — ao3c06461_si_001.pdf [file ao3c06461_si_001.pdf]
